# Identifying and mitigating batch effects in whole genome sequencing data

**DOI:** 10.1186/s12859-017-1756-z

**Published:** 2017-07-24

**Authors:** Jennifer A. Tom, Jens Reeder, William F. Forrest, Robert R. Graham, Julie Hunkapiller, Timothy W. Behrens, Tushar R. Bhangale

**Affiliations:** 1 Bioinformatics and Computational Biology Department, Genentech Inc, 1 DNA Way, South San Francisco, CA 94080 USA; 2 Human Genetics Department, Genentech Inc, 1 DNA Way, South San Francisco, CA 94080 USA

**Keywords:** Whole genome sequencing, Genotyping, Genome-wide association studies, Batch effects

## Abstract

**Background:**

Large sample sets of whole genome sequencing with deep coverage are being generated, however assembling datasets from different sources inevitably introduces batch effects. These batch effects are not well understood and can be due to changes in the sequencing protocol or bioinformatics tools used to process the data. No systematic algorithms or heuristics exist to detect and filter batch effects or remove associations impacted by batch effects in whole genome sequencing data.

**Results:**

We describe key quality metrics, provide a freely available software package to compute them, and demonstrate that identification of batch effects is aided by principal components analysis of these metrics. To mitigate batch effects, we developed new site-specific filters that identified and removed variants that falsely associated with the phenotype due to batch effect. These include filtering based on: a haplotype based genotype correction, a differential genotype quality test, and removing sites with missing genotype rate greater than 30% after setting genotypes with quality scores less than 20 to missing. This method removed 96.1% of unconfirmed genome-wide significant SNP associations and 97.6% of unconfirmed genome-wide significant indel associations. We performed analyses to demonstrate that: 1) These filters impacted variants known to be disease associated as 2 out of 16 confirmed associations in an AMD candidate SNP analysis were filtered, representing a reduction in power of 12.5%, 2) In the absence of batch effects, these filters removed only a small proportion of variants across the genome (type I error rate of 3%), and 3) in an independent dataset, the method removed 90.2% of unconfirmed genome-wide SNP associations and 89.8% of unconfirmed genome-wide indel associations.

**Conclusions:**

Researchers currently do not have effective tools to identify and mitigate batch effects in whole genome sequencing data. We developed and validated methods and filters to address this deficiency.

**Electronic supplementary material:**

The online version of this article (doi:10.1186/s12859-017-1756-z) contains supplementary material, which is available to authorized users.

## Background

Recent reductions in the cost of whole genome sequencing [1] (WGS) have paved the way for large-scale sequencing projects [2]. The rapid evolution of WGS technology has been characterized by changes to library preparation methods, sequencing chemistry, flow cells, and bioinformatics tools for read alignment and variant calling. Inevitably, the changes in WGS technology have resulted in large differences across samples and the potential for batch effects [3, 4].

Genotyping arrays preceded WGS and were the standard assay for variant calling and genome-wide association studies (GWAS). Batch effects are well studied in the context of genotyping arrays [5–7] and often can be addressed using widely adopted quality control (QC) measures [8]. Standard QC of SNP array data involves excluding samples with high missingness, testing for differences in allelic frequencies between known batches, removing related individuals, and correcting for population structure and possibly batch effects via principal components analysis (PCA) [8, 9]. QC strategies proposed for exome sequencing (WES) include empirically derived variant filtering [10] and methods for removing batch effects in copy number variation calling [11, 12]. These algorithms rely on read depth and either singular value decomposition (SVD), principal components analysis (PCA), or a reference panel to normalize read depth and remove batch effects [11–13].

Batch effects in WGS come with the additional complexity of interrogating difficult to characterize regions of the genome, and common approaches such as the Variant Quality Score Recalibration (VQSR) step in GATK [14] and processing samples jointly using the GATK HaplotypeCaller pipeline fail to remove all batch effects. Factors leading to batch effects are ill-understood and can arise from multiple sources making it difficult to develop systematic algorithms to detect and remove batch effects. The optimal way to address batch effects would be through up-front study design [15]. For instance, sequencing both cases and controls in each sequencing run would be optimal [16]. One could then eliminate all calls crossing genome-wide significance after performing a GWAS with batch as phenotype. Following these lines, replication [17] and randomization would also go far in reducing the impact of batch effects. However, given the scale and cost required to procure and sequence samples, optimal study design is often not an option. This is particularly relevant when working within large consortia where controls may come from a single source (e.g. TOPMed [18]) and cases from many disease focused collections.

Given that no standardized algorithms or heuristics currently exist to identify or address the issue of batch effects in WGS, batch effects have generally been handled by adopting stringent QC measures. The Type 2 Diabetes Consortium [19] used a series of filters including setting sites with GATK genotype quality less than 20 to missing and eliminating any site with greater than 10 % missingness within each ethnicity, deviation from HWE, and differential call rate between cases and controls on a dataset that included WGS and WES data. This filtering eliminated 9.9 % of SNPs and 90.8 % of indels. Similarly, the UK10K consortium [20] removed any site found as significant after performing an association study with sequencing center as the phenotype. This, alongside additional QC measures, resulted in removal of 76.9 % of variants [21]. Removing repetitive regions of the genome (removes ~53% of the genome) [22] or using established high confidence regions such as genome in a bottle (removes ~26% of the genome) [23] are similarly stringent.

In addition to removing unconfirmed and likely spurious associations induced by batch effects, researchers must also determine that a batch effect exists. Identifying a method to detect batch effects that have an impact on downstream association analyses is crucial as researchers need to know upfront whether WGS datasets can be combined or if changes in sequencing chemistry will result in sequences that can no longer be analyzed together. This has been done with principal components analysis [24] for SNP array data or for WES using various summary metrics of the data (such as read count, base quality, etc.) [25]. Metrics such as the percent variants confirmed in 1000 genomes data [26] can be used to assess WGS data quality. Similarly, transition-transversion ratios (Ti/Tv) are known to range from 2.0–2.1 in genomic and 3.0–3.3 in exonic regions [14]. Deviations from these values can indicate poor data quality.

The powerful technique of haplotype inference has evolved orthogonal to the established approaches to correct for batch effects [27–29]. Haplotype blocks are used for applications as diverse as imputation, identifying positive selection, and estimating population diversity [30–32]. Haplotype blocks have the potential to aid with correcting for batch effects as they are used to detect genotype error [30] and correct for poor genotyping quality [33].

Large-scale WGS efforts are thriving, however few guidelines exist for determining whether a dataset has batch effects and, if so, what methods will reduce their impact. We address both these deficiencies and introduce new software (R package, genotypeeval, see Methods for additional details and web link) that can help identify batch effects. We demonstrate how to identify a detectable batch effect in WGS data via summary metrics computed using genotype calls, their quality values, read depths, and genomic annotations, followed by a PCA of these metrics. We describe our strategy to eliminate unconfirmed genome-wide significant associations (UGAs), which are likely enriched for spurious associations, induced by batch effects. Our aim was to develop filters that removed sites impacted by a detectable batch effect with high specificity so as not to eliminate a large number of variants genome-wide. The filters we developed do not remove all UGAs impacted by batch effects and come at the cost of a reduction in power of 12.5%, however when applied in conjunction with standard quality control measures (see Methods) they can substantially mitigate the impact of batch effects.

We recommend the following three-step combination of filters to reduce UGAs: 1) Use haplotypes to correct errors in genotypes, then remove associations no longer achieving genome-wide significance (GWS, *P* < 5E-8) following that correction, 2) Impose a differential genotype quality filter, and 3) Set genotypes with quality scores less than 20 to missing, then filter any site missing 30% or more of its genotypes (we refer to this filter as “GQ20M30”). Application of this three-step filter substantially reduced UGAs (SNPs by 96.1%, indels by 97.6%, and overall by 97.2%). When applied to data for an Age-Related Macular Degeneration (AMD) study without a detectable batch effect, these filters removed only a small number of variants genome-wide (type I error rate of 3%). An AMD candidate SNP analysis revealed that these filters reduced power by 12.5%. Finally, an independent Rheumatoid Arthritis (RA) dataset with a different known source of detectable batch effect confirmed our proposed filters were effective (reduced UGAs 89.8%).

## Results

### Descriptive statistics

We analyzed 1231 samples sequenced at approximately 30× average depth using Illumina based WGS over a period of 5 years at various sequencing centers. Short reads were mapped to the genome using BWA-MEM [34] and variant calling was performed using GATK best practices [35]. All samples were jointly genotyped with GATK HaplotypeCaller. For each sample we computed various summary metrics based on the GATK genotype calls, genotype quality (GQ), read depth, and genomic annotations e.g. coding/non-coding. The goal of this initial analysis was to identify metrics that enable detection of batch effects.

**Fig. 1:**
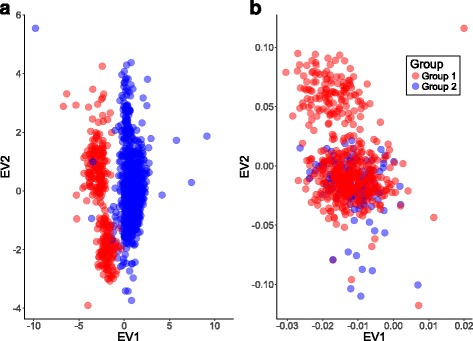
A detectable batch effect was apparent in PCA of relevant quality metrics calculated using the gVCF (**a**). The standard GWAS PCA performed using 250,000 common SNPs did not reveal this batch effect (**b**). Quality metrics included in the PCA in (**a**) include percent of variants confirmed in 1000 genomes (phase 1, high confidence SNPs) [26], mean genotype quality, median read depth, transition transversion ratio in non-coding regions, transition transversion ratio in coding regions, and percent heterozygotes. Group 1 here refers to samples sequenced in 2010–2012 and Group 2 to samples sequenced in 2013 and 2014

The scatterplot of the first two eigenvectors generated from PCA of key quality metrics (%1000 g, Ti/Tv in coding and non-coding regions, mean genotype quality, median read depth, and percent heterozygotes) clearly revealed a batch effect (Fig. 1a). Similar to [36] we did not observe this delineation in the standard GWAS PCA plot generated using genotypes at 250,000 common SNPs across the genome (Figure 1b). We defined a detectable batch effect in this study to be the existence of well-delineated groups determined by PCA of key quality metrics of sequencing data. We have implemented the methods to compute these metrics in the R package genotypeeval that can aid researchers in assessing the potential for batch effects when combining datasets from different sources.

This detectable batch effect could not solely be attributed to vendor, library preparation, sequencing chemistry, or size exclusion step (Additional file 2: Table S1) as none of these variables solely explained the differences between group 1 and group 2. It is likely that PCR-free versus PCR library preparation and sequencing center played a key role in creating this detectable batch effect, similar to [36], as we found clear separation in PCA visualizations of quality metrics by these variables (Additional file 1: Figure S1). We found the two groups were best explained using year of sequencing so designated samples sequenced in years 2010, 2011, and 2012 as group 1 (*N* = 918 samples) and samples sequenced in years 2013 and 2014 as group 2 (*N* = 313 samples).

We next explored in detail the six quality metrics used in our PCA decomposition (Table 1, Additional file 1: Figure S2, Additional file 2: Table S2). While read depth and GATK genotype quality (GQ) were comparable between the two groups (Table 1, Additional file 2: Table S2), metrics based on transition-transversion ratio (Ti/Tv), heterozygous calls, and percent of variants confirmed in 1000 genomes (%1000 g) showed highly statistically significant differences (Table 1, Additional file 2: Table S2).

**Table 1:** Descriptive metrics of 1231 whole genome sequences by batch

Variable Mean (SD)	Group 1	Group 2	*p*-value^*a*^
N	918	313	
GATK Genotype Quality	91.47 (2.72)	90.77 (3.57)	NS
Median Read Depth	33.65 (4.69)	35.39 (6.81)	NS
Ti/Tv in Non Coding Regions	2.01 (0.012)	1.95 (0.019)	< 0.0001
Ti/Tv in Coding Regions	2.99 (0.053)	2.90 (0.032)	< 0.0001
% Confirmed in 1000 Genomes	81 (0.87)	77 (0.76)	< 0.0001
Percent Heterozygote	7.5 (0.48)	8.2 (0.45)	< 0.0001

Group 1 and Group 2 refer to two different groups detected via a visualization of eigenvectors from a PCA of metrics derived from the gVCF files

*GATK* Genome Analysis Toolkit, *Ti/Tv* transition transversion ratio, *NS* not significant

The means of each variable are reported along with the standard deviation in parenthesis

^a^Differences between the two groups were assessed using the Wilcoxon Rank Sum Test, two-sided alternative, with a Bonferroni adjustment for multiple tests

To test the hypothesis that only particularly difficult-to-sequence regions of the genome were subject to batch effects, we computed our metrics after removing repeat-masked regions [22] (53.02% of genome), segmental duplications [37] (13.65%), self-chain regions [37] (6.02%), centromeres (2.01%), ENCODE blacklist [38] (0.39%), or low-complexity regions (0.21%). PCA plots of our quality metrics re-computed after filtering out the difficult to assay regions still clearly revealed detectable batch effects (Additional file 1: Figure S3). We again examined the metrics underlying the PCA plot by performing a Wilcoxon-Rank Sum test comparing group 1 and group 2 post-filtering (Additional file 1: Figure S4, Additional file 2: Table S2). Removing all repeat-masked regions narrowed the difference in %1000 g between groups from 4% to 1.8%, however %1000 g between groups was still statistically significant (*p*-value <2E-16). Removing smaller regions of the genome had only a modest effect on %1000 g and affected both groups similarly as the difference in %1000 g between the two groups remained between 3 and 4 %. Masking difficult regions had little influence on the GQ. There was some impact on median read depth – after filtering out GQ less than 90 the median read depth metric was significantly different between groups (*p*-value = 0.0004). Filtering did not impact the Ti/Tv ratio metrics in non-coding or coding regions. Differences between groups for the percent heterozygous metric improved after repeat masked regions were removed (*p*-value 0.823) but remained unchanged for all other filters. This analysis suggested that filtering variants based only on excluding difficult regions was not an effective strategy.

### Mitigating batch effects via filtering

Large-scale genome-wide association studies using SNP array based data often combined cases and controls obtained from different sources [39–41] and this practice continues with WGS based data [19, 20]. Rigorous QC of SNP array based data reduced batch effects in this setting. The sensitivity of WGS technology to differences in library preparation, sequencing chemistry, etc. makes it markedly susceptible to batch effects, however no standard set of guidelines for QC of WGS has been established. We therefore considered this challenging scenario by performing a GWAS comparing 642 samples from group 1 and 173 from group 2 with group as a phenotype (Batch GWAS). These samples did not differ in terms of their disease phenotype and at these sample sizes no GWS associations were expected in this analysis. To eliminate another potential source of batch effect -- an algorithmically induced effect from read alignment and genotype calling, the short read data for these samples were analyzed using the same bioinformatic pipeline and the samples were jointly genotyped using GATK HaplotypeCaller. In addition, QC steps used in standard SNP-array GWAS were applied (see Methods). Despite this, 1901 SNPs and 5469 indels (Additional file 1: Figure S5) had a genome-wide significant association. We refer to these as unconfirmed genome-wide significant associations (UGAs). These UGAs were distributed throughout the genome and were not filtered by applying QC procedures such as HWE, high missingness by site, or masking out difficult to sequence regions. Genomic inflation (λ_GC_) was high for this study at 1.07 as was genomic inflation corrected for small sample size (λ_1000_) at 1.25 (Additional file 1: Figure S6). An analysis stratified by minor allele frequency (MAF) of sites revealed genomic inflation was highest for low frequency variants (MAF 1% to 5%, λ_GC_ = 1.05, λ_1000_ = 1.19, Additional file 1: Figure S7). Stratification by GC content of sites, calculated using a 25 base pair window surrounding the association, showed genomic inflation was highest for low GC content (GC < = 20%, λ_GC_ = 1.14, λ_1000_ = 1.51, Additional file 1: Figure S8).

**Fig. 2:**
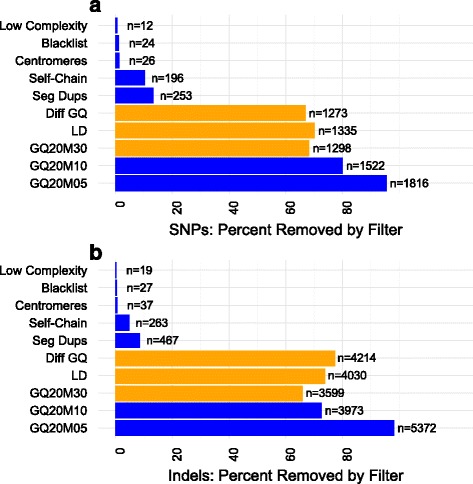
Filtering unconfirmed genome-wide significant associations (UGAs) from the Batch GWAS. Percent (and number, n) of the 7370 UGAs (1901 SNPs and 5469 indels) removed by each filter for (**a**) SNPs and (**b**) indels. In yellow are the filters we recommend and in blue are other filters we tested

**Fig. 3:**
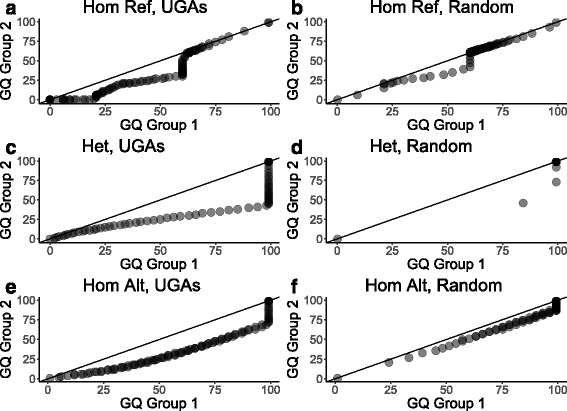
Quantile-quantile plots revealed differences in genotype quality (GQ) distributions. Hom Ref, homozogyous reference (**a**,**b**); Het, heterozygotes (**c**,**d**); Hom Alt, homozygous alternative (**e**,**f**); UGAs, sites with *p*-value <5E-8 in the Batch GWAS; Random, comparable set of sites with *p*-value >5E-8 in the Batch GWAS. Note that in (**d**) most points overlap the single darkest point on the plot

The above scenario, while challenging, is likely to be encountered frequently in practice. We studied a number of filters that removed these UGAs in an efficient manner i.e. without eliminating too many of the variants across the genome (Fig. 2, Additional file 2: Table S4, S5). Linkage Disequilibrium (LD) can be used to correct genotyping errors [42] where a genotype incompatible with the surrounding haplotype is corrected. In the LD filter, a variant was removed if the association test based on the corrected genotypes obtained using Beagle [29] was not GWS. This eliminated 1335 out of 1901 or 70.22% of UGA SNPs. Based on the observation that GQ distributions at UGAs were often substantially different between the two batches, a pattern not seen in randomly selected sites that were not genome-wide significant in the Batch GWAS (Fig. 3), we developed the differential GQ filter (see Methods). Based on simulated data (see Methods), the differential GQ filter had 80% power with a GQ difference of 15 between groups and sample size of 500 per group (Additional file 1: Figure S9). After we applied the differential GQ filter, we had 566 SNP and 1439 indel UGAs. On its own, the differential GQ filter eliminated 1273 or 66.96% of UGA SNPs. Finally we used the GQ20M30 filter where first, genotypes with GATK GQ score less than 20 were declared missing and then sites with missing genotype rate greater than 30 % were removed. This left us with 74 UGA SNPs. Almost all UGA SNPs were removed with more stringent filtering. A stringent GQ20M05 filter on its own eliminated a comparable number of SNPs as our proposed filtering (1816 SNPs or 95.53% of the SNPs filtered, 85 SNPs remained). In combination with our proposed filtering, the GQ20M05, LD, and differential GQ filters left only 16 UGA SNPs. Similarly, a GQ20M10 filter in combination with our proposed filters left only 38 UGA SNPs (Additional file 2: Table S5).

While methods for calling indels from WGS data are not as reliable as methods for calling SNPs [43], our approach filtered most UGA indels (elimination of 97.6% of the 5469 UGA indels). The LD filter removed 4030 UGA indels (73.69%), the differential GQ filter removed an additional 1044 or 72.55% of the remaining 1439 UGA indels, and the GQ20M30 filter removed an additional 264 or 66.84% of the remaining 395 UGA indels leaving us with 131 out of the original 5469 UGA indels to assess. Again, the GQ20M05 filter on its own removed a comparable number of UGA indels (5372 out of 5469 or 98.23 %) and left 97 indels unfiltered. Using the GQ20M05 filter in conjunction with the LD and differential GQ filters left 19 UGA indels. The GQ20M10 filter in combination with our filters left 97 UGA indels.

We also evaluated whether difficult to assess regions (repeat masked, low complexity, centromeres, ENCODE blacklist, segmental duplications and self chain regions) added to the above-described filters. Most of these annotations removed only a few sites after our proposed filters were applied (see Additional file 2: Table S5). The most effective annotation filter, repeat masking, removed about half the remaining 74 UGAs.

We saw modest improvement in the genomic inflation factor from 1.07 (λ_1000_ = 1.25) to 1.06 (λ_1000_ = 1.22, Additional file 1: Figure S6, Additional file 2: Table S3, S6). We found the most substantial improvement in genomic inflation factor when stratified by minor allele frequency (MAF) for low frequency variants (MAF of 1 to 5%) from 1.07 (λ_1000_ = 1.24) to 1.05 (λ_1000_ = 1.19, Additional file 2: Figure S10). A similar stratification by GC content showed the most improvement for low GC (GC < = 20%) where genomic inflation factor improved from 1.17 (λ_1000_ = 1.63) to 1.14 (λ_1000_ = 1.51, Additional file 2: Figure S11). The overall percent of UGAs filtered was 97.2%. When stratified by GC content, we found the highest percent of UGAs filtered (98.8%) for the sites with lowest GC content (GC < = 0.2). When stratified by minor allele frequency, the highest percent filtered was for the low frequency variants (MAF of 1 to 5%, 98.5% filtered, Additional file 2: Table S6).

**Fig. 4:**
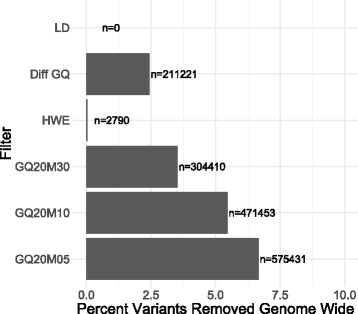
Performance of filters on an Age-Related Macular Degeneration (AMD) GWAS with no batch effect. Percent (and number, n) of variants removed genome-wide in an AMD GWAS with no batch effect where 8,636,121 unique sites and 8,791,425 variants (SNPs and indels) were analyzed

In the absence of batch effects, an effective filtering strategy will eliminate a relatively small number of variants. We assessed the impact of our strategy by performing a genome-wide analysis comparing 1218 cases of Age-related Macular Degeneration (AMD) and 250 controls from the same batch. These samples had the same vendor, chemistry, and were jointly genotyped in a single run. We verified the absence of a batch effect by performing a PCA on the quality metrics as described previously and saw no detectable batch effect as the samples were completely overlapping (Additional file 1: Figure S12). In this AMD GWAS with no batch effect, we had 220 significant associations (at variants in LD with each other) that we refer to as confirmed associations [44] as these fell in the two well-known AMD loci CFH and ARMS2-HTRA1 [42]. With our sample size we had sufficient power to detect association (see power calculation, Additional file 2: Table S7) at these two (out of 19) previously known AMD loci. In addition, we detected a GWS association at APOE as our controls were enriched for Alzheimer’s cases. Alzheimer’s cases are older on average and are unlikely to be carriers of variants for AMD. We had a handful of UGAs (16 SNPs, 31 indels). Most UGAs were in repeat masked regions (16 SNPs, 24 indels). Interestingly 15 of the 16 UGA SNPs were eliminated by the differential GQ filter (Additional file 2: Table S8). Genome-wide, we filtered a minimal number of sites with our batch effects specific filters (Fig. 4, Additional file 1: Figure S13, Additional file 2: Table S8). The LD filter did not impact any sites. The differential GQ filter removed 211,221 out of 8,636,121 variants or 2.4% of the variants. The GQ20M30 filter removed 3.4% (304,410) of variants, the GQ20M10 filter removed 5.5% (471,453) of variants, and the GQ20M05 removed 6.6% (575,431) of variants. Given that the GQ20M10 filter removed 2% more of the variants genome-wide than the GQ20M30 filter and it did not filter out a large proportion of additional UGAs, we recommend the GQ20M30 filter. The genomic inflation factor prior to filtering was 1.02 (λ_1000_ = 1.04) and post filtering was 1.01 (λ_1000_ = 1.02), reflecting a slight improvement in genomic inflation (Additional file 1: Figure S14).

We next performed an analysis to verify that in presence of batch effects, our filtering strategy did not negatively impact confirmed associations. To this end, we analyzed 1252 cases of Age-related Macular Degeneration (AMD) and 678 controls with a detectable batch effect (Additional file 1: Figure S15) at SNPs spanning 1 Mb around 19 known AMD loci [44] (Additional file 2: Table S7; see Methods for power analysis). In the AMD candidate SNP analysis with batch effect, we examined 19 confirmed associations. Due to sample size, we lacked the power (Additional file 2: Table S7) to detect a significant association at the majority of these SNPs. We therefore examined if our method filtered any of the variants or changed the *p*-values from significant to non-significant. The detectable batch effect in the AMD candidate SNP analysis was quite pronounced as it was also detected in the PCA of the 250,000 common SNPs (Additional file 1: Figure S15). After applying standard QC filters (see Methods), we retained data on 16 out of the 19 known loci. The stringent GQ20M05 filter removed SNPs from 12 of these known AMD loci (Table 2). However, the GQ20M30 filter removed none, the LD filter changed none of the *p*-values from significant to non-significant or vice versa, and the differential GQ filter removed only two of the known loci. These results indicated that our filtering strategy specifically targeted batch effects and as a result it retained more sites overall and most confirmed associations. The more stringent GQ20M05 filter removed the majority of these known AMD associations.

**Table 2:** Retaining confirmed AMD associations in a candidate SNP analysis when batch is completely confounded with AMD status

CHR	Position^a^	*p*-value	Percent missing	GQ20M05	GQ20M30	Diff GQ	LD corrected *p*-value
1	196,710,325	0.002199	4.928	NF	NF	NF	0.002122
3	64,719,689	NS	5.027	F	NF	NF	NS
3	99,762,695	NS	5.027	F	NF	NF	NS
6	43,858,890	NS	6.57	F	NF	F	NS
6	116,122,572	NS	6.471	F	NF	NF	NS
8	23,225,458	NS	6.72	F	NF	NF	NS
9	99,146,083	NS	5.475	F	NF	NF	NS
10	122,454,932	1.60E-05	0.6969	NF	NF	NF	1.79E-05
13	31,245,188	NS	5.625	F	NF	NF	NS
14	68,318,360	NS	5.226	F	NF	NF	NS
15	58,396,268	NS	5.625	F	NF	NF	NS
16	56,963,321	NS	3.833	NF	NF	F	NS
19	6,718,376	NS	3.534	NF	NF	NF	NS
19	44,919,689	2.30E-21^b^	6.67	F	NF	NF	3.16E-21
22	32,663,679	NS	6.521	F	NF	NF	NS
22	38,080,269	NS	6.272	F	NF	NF	NS

*NF* is not filtered, *F* is filtered, GQ20M05 filter, filter sites with more than 5% missingness after setting genotypes with GQ < 20 to missing; GQ20M30 filter, filter sites with more than 30% missingness after setting genotypes with GQ < 20 to missing

*Diff GQ*, differential genotype quality filter, *LD* linkage disequilibrium, *NS* not significant in candidate SNP analysis at Bonferroni adjusted significance level: 0.05/16 = 0.00312

^a^Sites are reported in GRCh38 coordinates

^b^We detect APOE because our controls are enriched for Alzheimer’s cases

Finally, we analyzed another independent dataset with a suspected large batch effect to evaluate the effectiveness of our method. This was 30× WGS data Rheumatoid Arthritis cases sequenced at a single vendor and jointly genotyped. A detectable batch effect was expected for this data as a known change in sequencing chemistry (Additional file 2: Table S1) was introduced between 2015 (Chemistry 1, *n* = 770) and 2016 (Chemistry 2, *n* = 1528). Indeed, after performing PCA using our quality metrics as described above on these samples, we observed a detectable batch effect explained by chemistry (Additional file 1: Figure S16a) that was not evident in the standard GWAS PCA of 250,000 common SNPs (Additional file 1: Figure S16b). Performing a GWAS with sequencing chemistry as the phenotype (RA Batch GWAS), we observed 381,139 UGAs (46,841 SNPs and 334,298 indels), and a genomic inflation factor of 1.4 (λ_1000_ = 1.39, Additional file 1: Figure S17, Additional file 2: Table S9).

We found in this dataset that again, there was no enrichment of UGAs in difficult to sequence regions of the genome, except in the case of repeat regions that contained 83.3% of the UGA indels and 86.9% of the UGA SNPs (Additional file 2: Table S10). The differential GQ filter was the most effective filter in this dataset, removing 87.3% of UGAs overall (86.3% of SNPs and 87.4% of indels, Additional file 2: Table S11). The combination of LD, GQ20M30, and Differential GQ filter removed 89.8% of UGAs overall (90.2% of SNPs and 89.8% of indels). We saw a drop in λ_GC_ from 1.4 to 1.2 (λ_1000_ from 1.39 to 1.2, Additional file 1: Figure S17, Additional file 2: Table S9).

## Discussion

While sequencing costs are decreasing, many thousands of samples are necessary to have sufficient power to identify novel variants associated with common complex diseases [45]. In order to collect enough cases for diseases, multiple groups often work collaboratively by contributing samples to a consortium. In order to analyze these cases an even greater number of controls are desired [46]. Thus the need to combine samples that have been processed independently is clear, as is the unavoidable introduction of batch effects. These batch effects are subtle and simple filtering e.g. removing variants in “difficult regions” is ineffective. We found that changes in sequencing chemistry related to PCR versus PCR-free workflows strongly contributed to the detectable batch effects in both the Batch GWAS and the RA Batch GWAS.

Our R package, genotypeeval can process genotypes stored in gVCF (see Methods) or VCF files [26] and computes 46 metrics selected to assess the quality of WGS data. We ran this package in parallel in an hour on a single thread using 40 Gb of memory per sample.

Our initial efforts to perform association analyses in the presence of batch effects revolved around masking difficult to sequence regions, however we found this approach ineffective. In our Batch GWAS we did not see enrichment for UGAs in the repeat regions. This observation led us to develop and validate site-specific filters that target UGAs that arise from batch effects. We pursued the differential GQ filter because we observed in multiple datasets a systematic shift in GQ when sequencing chemistries changed. The LD filter was effective because the factors that led to batch effects are largely expected to be independent of the local LD structure. Thus the genotypes at UGA variants were not compatible with the surrounding haplotypes and these genotypes were corrected. The GQ20M30 filter addressed a need for a minimal quality threshold on the site. While we explored increasing the stringency on this filter, we found 30% missingness to be a reasonable tradeoff between retaining sites and removing batch effects. Therefore we recommend, in addition to standard GWAS QC, the LD filter, differential GQ filter, and the GQ20M30 filter while bearing in mind that these filters will reduce power to detect confirmed associations. We have also found that these filters may not be effective in the case of a severe batch effect – in this instance it may be necessary to adapt a more stringent filter such as GQ20M05, which will result in further reduction of power.

Our method to eliminate spurious calls can be applied when case and control status is completely confounded with batch. However, in this report we have focused on common variants. Effective strategies for rare variants still need to be addressed, though new algorithmic approaches are being developed [21]. We describe here an approach for minimizing batch effects when analyzing data from Illumina short-read sequencing, processed using BWA-MEM and GATK HaplotypeCaller. Further work is needed to assess the best way to cope with batch effects when using other sequencing technologies and variant calling pipelines. Another limitation of our investigation was our inability to examine read depth (see Methods) at a given site as this has been found to be a key contributor to artifacts in variant calling [47]. Our work focused on real data as a large number of factors contribute to batch effects in WGS data and any assumptions made to simulate batch effect data will likely be inadequate and at times inappropriate when working with real datasets. This was also a limitation of our investigation as we used only a single test dataset (the Batch GWAS) to develop our methods and two independent methods to validate on – the RA Batch GWAS for sensitivity and the AMD No Batch GWAS for specificity. Additionally, while the total sample size in our Batch GWAS was 1231 samples, the uneven distribution of samples (918 in Group 1 and 313 in Group 2) means we were limited in our power to detect as many associations due to batch effects than if our samples were evenly distributed between groups.

A final limitation of our methodology is that we have focused mostly on filtering out GWS associations and therefore we were much more effective in filtering in the genome-wide significant range of *p*-values than overall. This was reflected in the small gains in genomic inflation factors post filtering (eg in the Batch GWAS from 1.07 to 1.06) despite the large percent of UGAs filtered (97.2% in the Batch GWAS). We chose to focus on GWS unconfirmed associations since practically scientists want to prioritize these for further research and validation.

## Conclusions

We showed that the quality metrics we developed can determine whether a batch effect exists within a dataset and released software that allows researchers to quickly assess the quality of their sequencing data. After testing existing WGS filters, we recommended our filtering strategy which combines (1) an LD filter, (2) differential GQ filter, and (3) GQ20M30 filter. This combination of filters removed 97.2% of the unconfirmed genome-wide significant associations in the Batch GWAS and 89.8% in the RA Batch GWAS. An AMD GWAS with no batch effect featured a Type I error rate of 3% and an AMD candidate SNP analysis revealed a reduction in power of 12.5% as 2 out of 16 confirmed AMD associations were filtered.

Batch effects in WGS data are not well understood and perhaps because of this, we were not able to find an existing method or develop a novel method that removed all sites impacted by batch effects without impacting the power to detect true associations. While we focused on creating targeted filters that removed a small percent of the genome, in practice these need to be used in conjunction with standard quality control measures (for example removing sites out of Hardy-Weinberg equilibrium), which can result in very stringent filtering. In the case of a severe batch effect, such as the chemistry change present in the RA Batch GWAS, more stringent filtering was necessary even after applying standard quality control and our proposed filters as almost 40,000 UGAs remained after filtering. In order to fully address batch effects, disentangling the impact of changes in sequencing chemistry and bioinformatics processing on association analysis will be necessary.

Batch effects will arise as independent groups attempt to combine sequencing data generated and processed from different sources – this collaboration is necessary particularly to attain power to detect new disease-associated variants. Large-scale resources are spent by research, industry, and government organizations creating databases that cannot easily be merged. Our experiments and tools will help researchers integrate this rich mine of genetic data.

## Methods

### Samples and sequencing

Samples were collected under appropriate consent approved by the Western Institutional Review Board through multiple ongoing collaborations. For all samples DNA was extracted from whole blood. The size exclusion step was performed using gel or SPRI and library preparation methods varied between different Illumina techniques: PCR-based, PCR-free, and PCR-plus. Thus multiple parameters varied between years and vendors and no single parameter was found to correspond to the observed batch effect in our samples. Sequencing was conducted on Illumina X 10 and HiSeq machines between the years of 2010 through 2016 using Illumina, Beijing Genomics Institute (BGI), DeCODE, Broad Institute (Boston), and Human Longevity Inc. (HLI) as sequencing vendors (Additional file 2: Table S1). All sequencing involved generating paired-end reads with the target average genome coverage of 30×.

All samples were processed using the same sequence alignment and variant calling pipeline. Short read data were aligned to GRCh38 using BWA-MEM [34] and the resulting alignments (bam files) were processed using GATK best practices [35] to first generate per-sample genome-wide genotype calls (gVCF files). A single multi-sample VCF was then created by jointly genotyping all gVCF files using GATK HaplotypeCaller. The data was analyzed using GATK version 3.4 which did not accurately report read depth in the final VCF due to a local reassembly step (see http://gatkforums.broadinstitute.org/gatk/discussion/comment/36686#Comment_36686). During variant calling GATK HaplotypeCaller performed a local de-novo assembly of the reads. Due to this, the effective read depth at the time of variant calling could be different than the read depth in the original alignments and the read depths in the original alignments were reported in the final VCF.

We developed a software package: genotypeeval freely available on Bioconductor as part of the R Project [48] to compute 46 metrics using gVCF files, including percent confirmed in 1000 genomes, Ti/Tv in coding and non-coding regions, number of heterozygous calls in self-chain regions, etc. Metrics identified as relevant to batch effects qwew described in this manuscript.

### Masking difficult to sequence regions

Difficult to sequence regions were assessed using the following annotation tracks: 1. repeat-masked regions [22], 2. low-complexity regions within the repeat-masked regions, 3. centromeres, 4. the ENCODE blacklist, [38] 5. self-chain regions from UCSC [49] and 6. segmental duplications from UCSC [50]. Where appropriate, tracks with coordinates in the older build hg19, were lifted over to GRCh38 using the liftover tool in the R package, rtracklayer [51].

### Power calculation

The 19 known AMD SNPs sites from [44] were evaluated to determine which SNPs we had sufficient power in our GWAS experiments to detect. The odds ratios and allele frequencies were obtained from [44] and evaluated for our AMD GWAS with no batch effect (1218 cases and 250 controls) as well as the AMD candidate SNP analysis with batch effect (1252 cases and 678 controls). Power calculations were done using CaTS [52] assuming an additive model and genome-wide significance level of 5xE-8.

### GWAS analyses

PLINK 1.9 [53] was used to run GWAS analysis after multi-allelic sites were removed. QC steps included removing sites with missing genotype rate greater than 50% and removing samples with greater than 20% missing genotype rate. Low minor allele frequency sites (less than 1%) were removed and sites out of Hardy-Weinberg equilibrium in controls (or group 1) alone were removed (*p*-value <1xE-5). Close relatives and individuals related to multiple individuals (potential sample contamination) were removed. Association analysis was performed using logistic regression of phenotype on additively coded genotypes, and the first five eigenvectors from PCA analysis [54] were included as covariates to correct for population structure. Sites with *p*-value <5xE-8 were considered genome-wide significant (GWS).

The Batch GWAS analysis used 815 subjects in total (642 in Batch 1 and 173 in Batch 2). GWAS as outlined above was performed and any GWS association was considered an unconfirmed genome-wide significant association (UGA) – this assumption was made because of the relatively small sample size and because there were no known confirmed associations for the phenotypes included in the sample. We identified 1901 UGA SNPs and 5469 UGA indels for a total of 7370 UGAs.

### Filters

#### GQ20Mx filter

Genotype calls with genotype quality score computed by GATK HaplotypeCaller less than 20 were set to missing. With the GQ20Mx filter, sites with greater than x% missing genotype rate were filtered. For example, in the case of the GQ20M10 filter, sites with greater than 10% missing genotype rate were filtered.

#### LD based genotype correction

The jointly genotyped VCF file generated by GATK was analyzed using Beagle Version 4.1 [29], to obtain LD corrected genotypes. The GWAS analysis as outlined previously was performed using the LD corrected VCF file. For example, a genotype incorrectly identified as a heterozygous is unlikely to be compatible with the surrounding haplotype block and will likely be corrected to a homozygous genotype prior to analysis. Therefore sites where genotypes were disproportionately and incorrectly called heterozygotes in a single batch will no longer be identified as GWS. Sites that were no longer GWS after using LD-corrected genotypes in the association test were filtered.

#### Differential GQ filter

Genotype qualities were dichotomized at GQ60. A chi square test with the variables batch (for example in the Batch GWAS, group 1 and group 2) and dichotomized GQ60 was used to test for differential genotype quality with a *p*-value cutoff of 1E-4. Homozygous reference, heterozygote, and alternative genotypes were tested independently at a given site and the site filtered if any of the three tests were significant.

Simulations to assess power were performed by drawing group 1 genotype quality scores from a continuous uniform distribution (X_1_ ~ Uniform(0,99)) and group 2 genotype quality scores from a continuous uniform distribution with added normal noise (X_2_ ~ Uniform(0,99) + Normal(mu, sigma)). Sigma was tested at 1, 5, and 10. Mu varied from 0 to 20 and sample size was tested at 250, 500, and 1000. The simulations were repeated 1000 times each.

## Additional files

Additional file 1: Supplemental Figs. S1-S17. (PDF 4953 kb)

Additional file 2: Supplemental Tables S1-S11. (PDF 112 kb)

Additional file 3: Sample level summary statistics and annotations calculated by genotypeeval. (CSV 102 kb)

## Abbreviations

% 1000 g: Percent confirmed in 1000 genomes
AMD: Age related macular degeneration
GATK: Genome Analysis Toolkit
GQ: Genotype quality
GWAS: Genome-wide association study
GWS: Genome-wide significant
LD: Linkage disequilibrium
QC: Quality control
Ti/Tv: Transition Transversion Ratio
UGA: Unconfirmed genome-wide significant association
WES: Whole exome sequencing
WGS: Whole genome sequencing
