# Supplementary material for: Identifying and mitigating batch effects in whole genome sequencing data
Source: BMC Bioinformatics. 2017 Jul 24;18:351. doi: 10.1186/s12859-017-1756-z (PMC5525370; doi:10.1186/s12859-017-1756-z)
Supplement: Supplementary file 1 — (PDF 4953 kb) [file 12859_2017_1756_MOESM1_ESM.pdf]

a

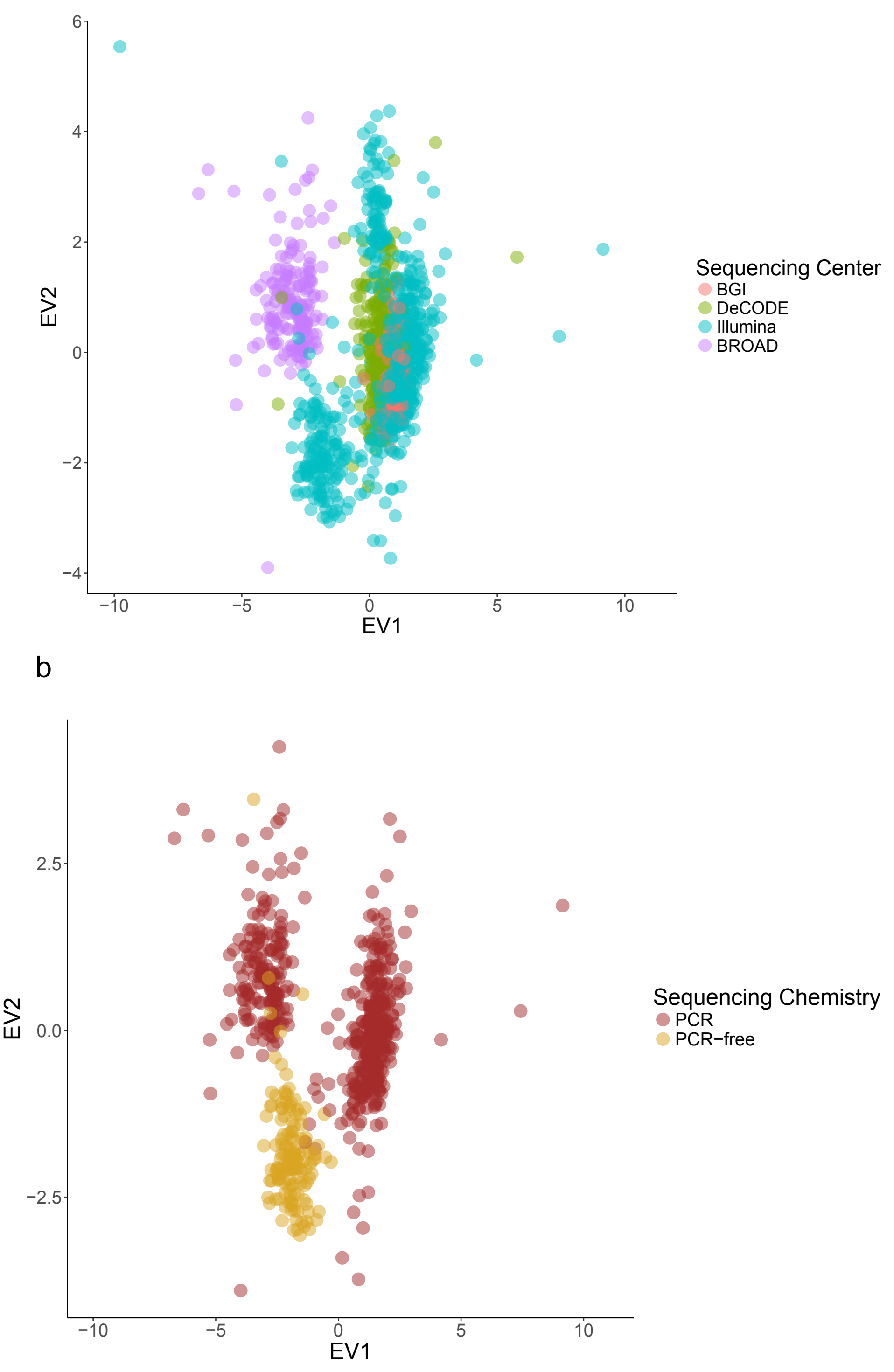

b

Figure S1. PCA plots of quality metrics for Batch GWAS. See Figure 1 for details of quality metrics. In a) are the first two eigenvectors plotted for PCA applied to summary statistics from genotypeeval colored by sequencing center. In b) is colored by sequencing chemistry (where information is available, n=645)

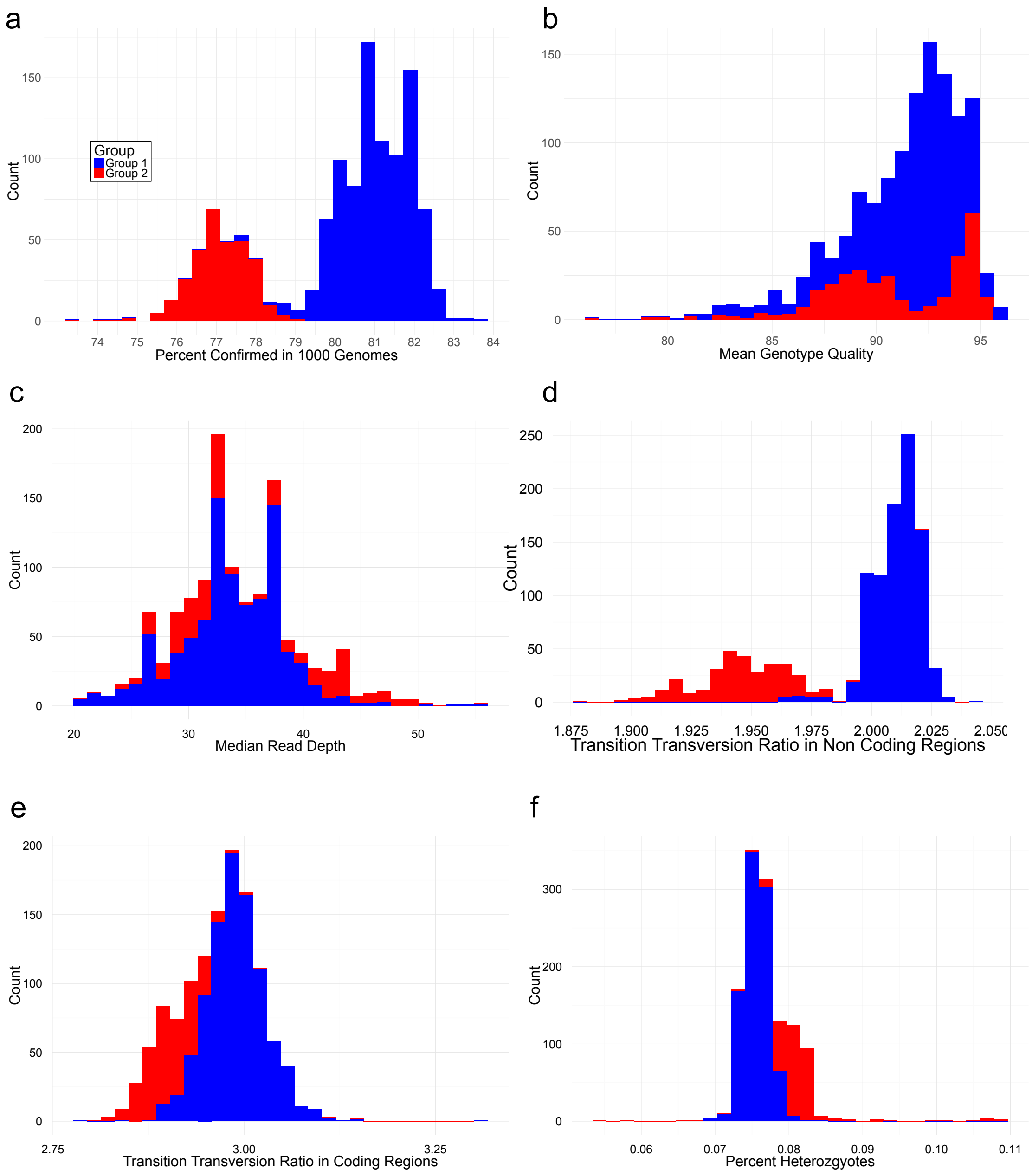

Figure S2. Histograms of summary metrics of interest by group. Group here refers to the batches within the batch GWAS samples which were defined as group 1: Sequenced in 2010-2012, and group 2: Sequenced in 2013 and 2014.

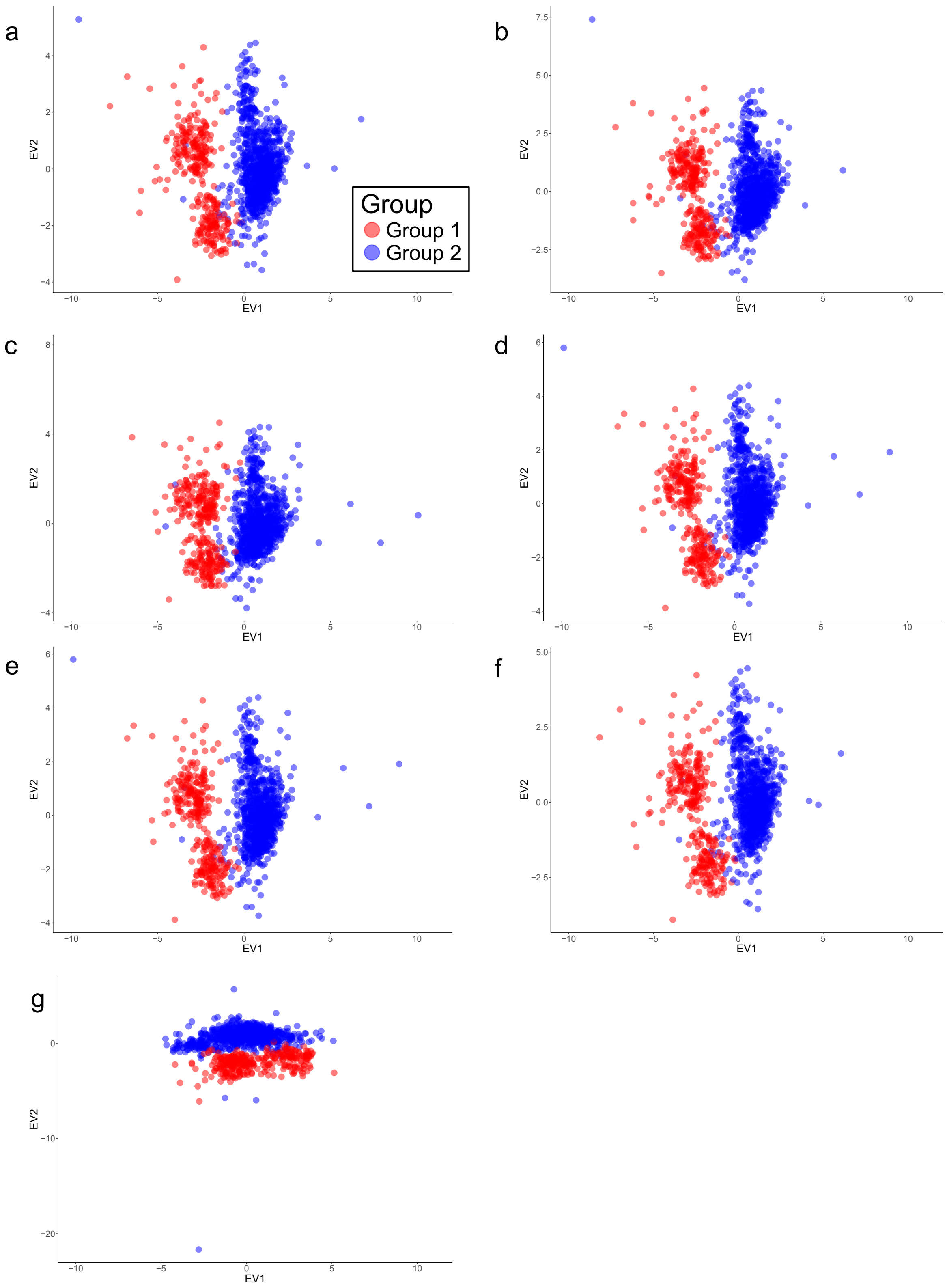

Figure S3. Detectable batch effect remains after filtering a) Self-Chain, b) Low Complexity, c) Centromeres, d) Blacklist, e) Genotype Quality < 90, f) Segmental Duplications, and g) Repeat masked regions. For details see Figure 1.

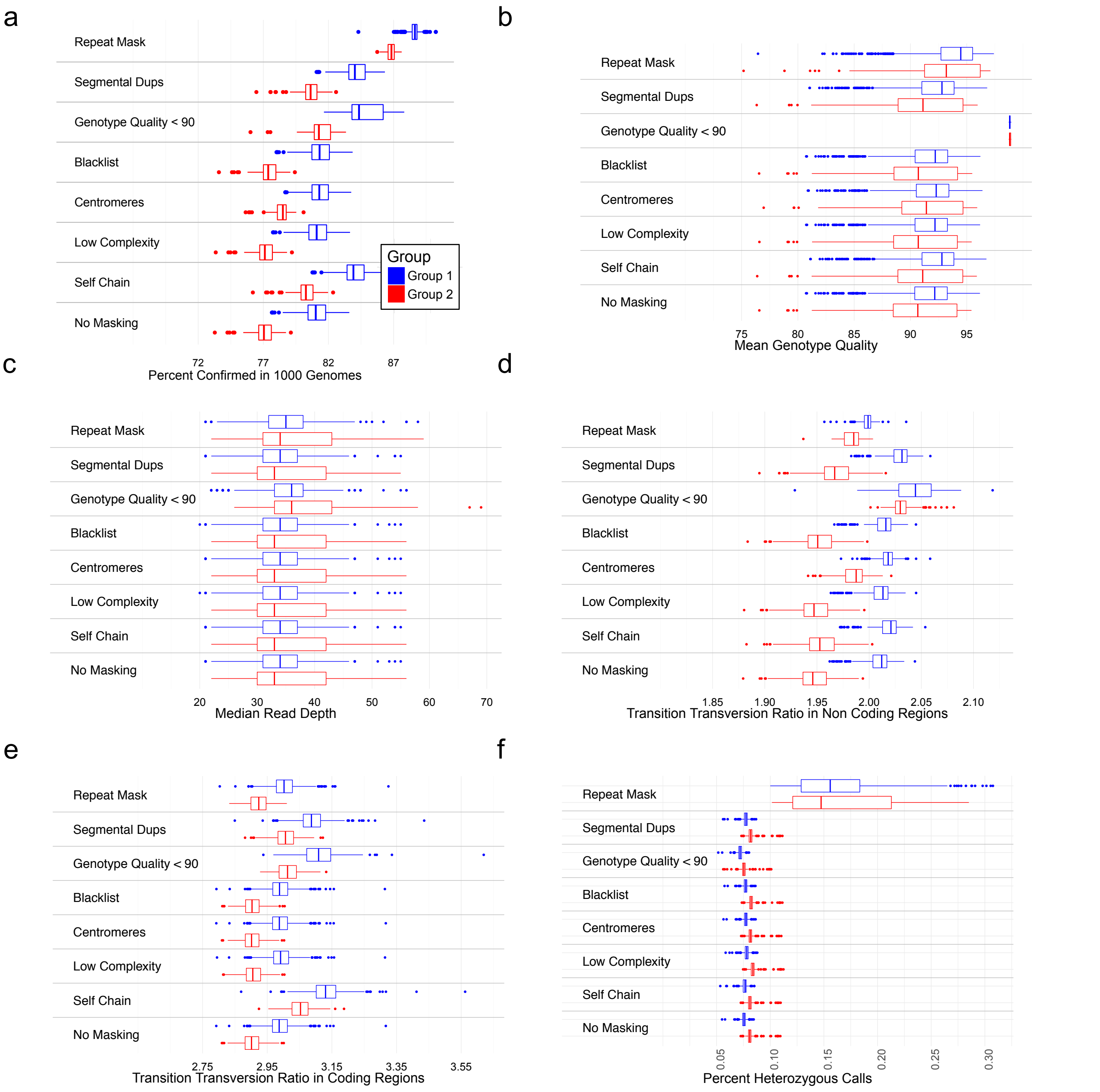

Figure S4. Mitigating batch effects by masking difficult regions was challenging as the batch effect remained post filtering. For annotation information see Methods.

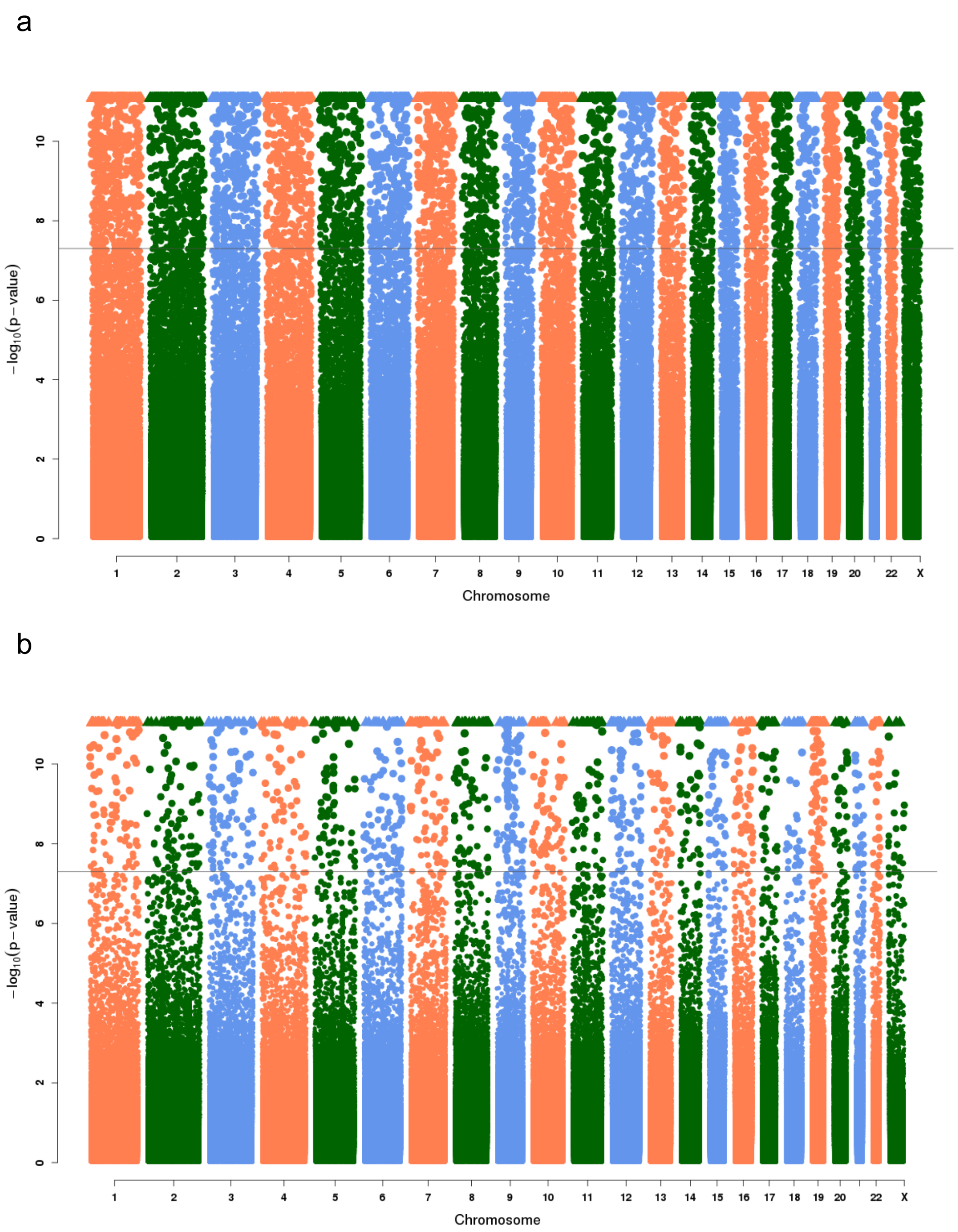

Figure S5. Manhattan plot from Batch GWAS. Group 1: n=740, sequenced in 2010, 2011, or 2012 and group 2: n=175, sequenced in 2013 or 2014. In (a) is all variants and (b) is SNPs only.

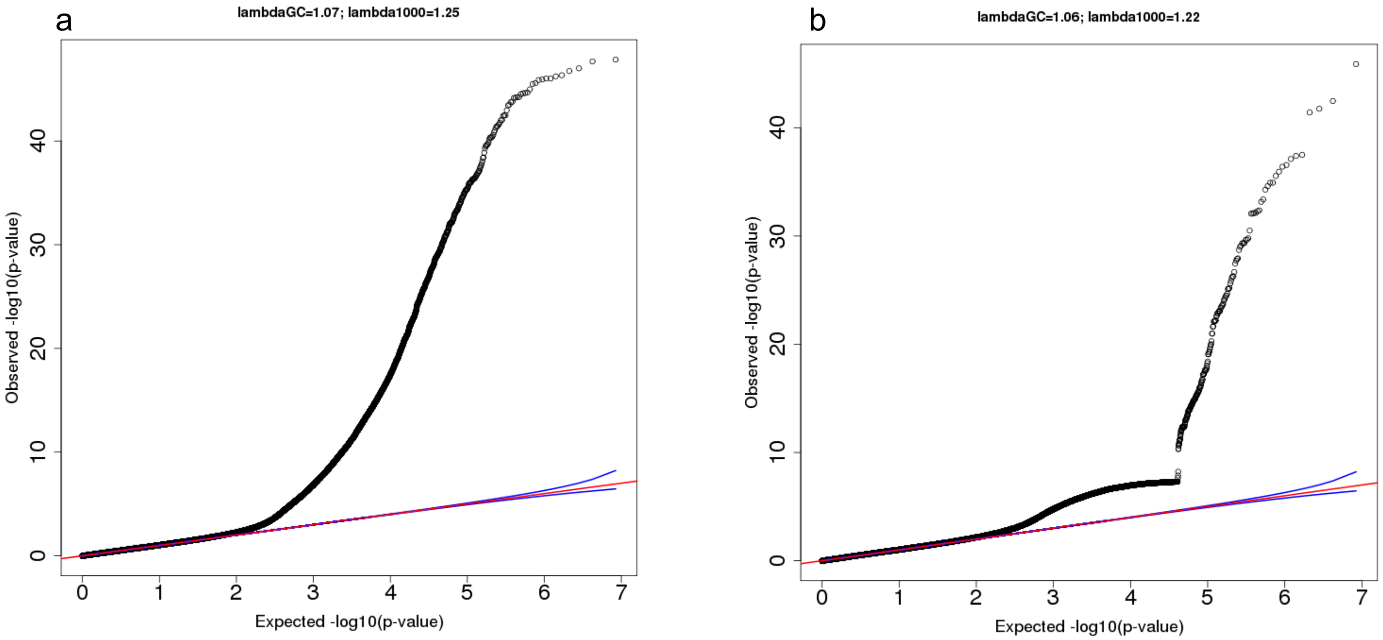

Figure S6. QQ plots for a) Batch GWAS and b) Batch GWAS post filtering. lambdaGC is genomic control, lambda1000 is genomic control with small sample correction, in red is the reference line and in blue are 95% confidence bands for the reference line.

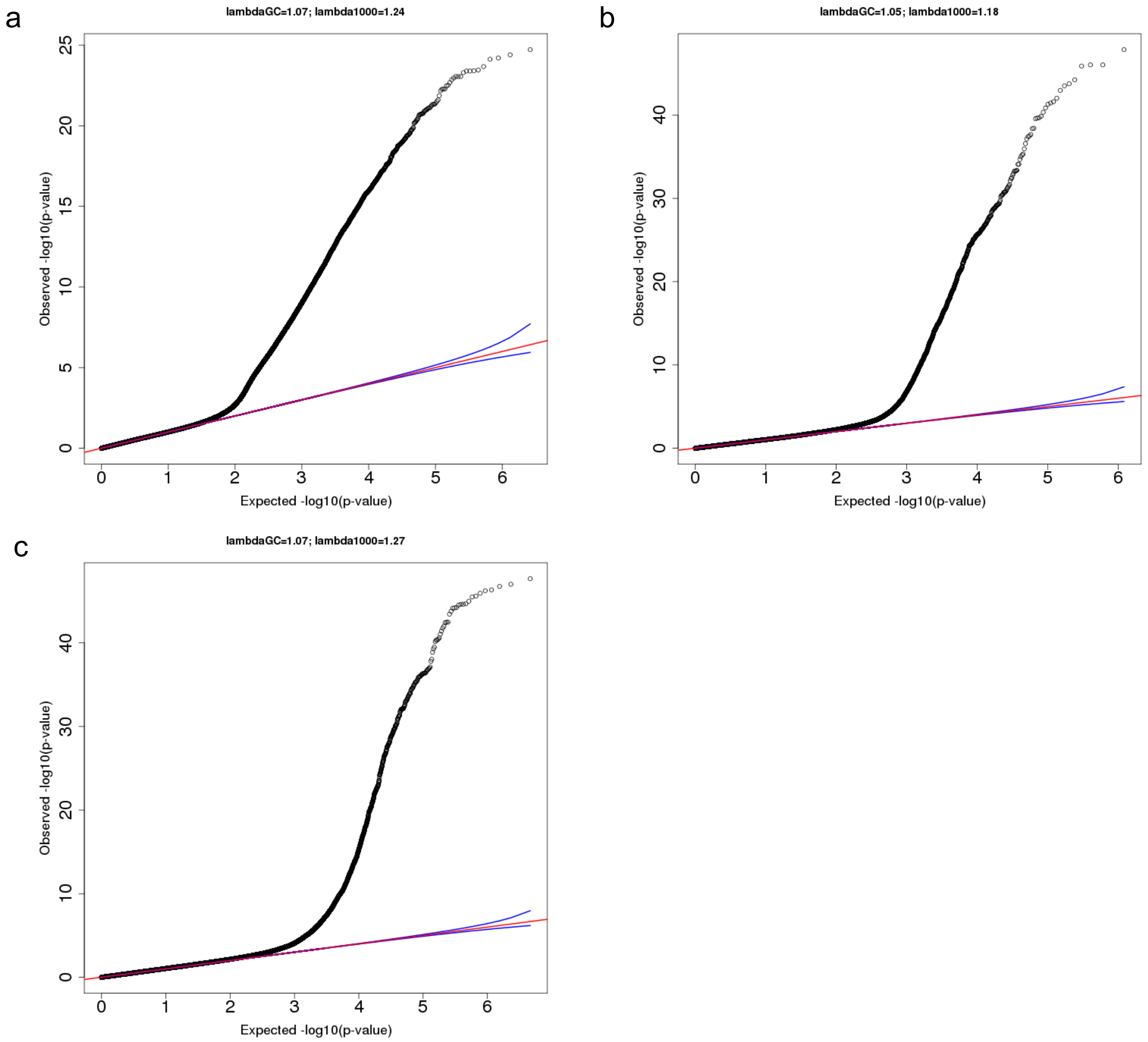

Figure S7. QQ plots for Batch GWAS stratified by MAF, a) is 0.01 <= MAF <= 0.05, in b) is 0.05 < MAF < 0.1, and c) is 0.1 < MAF <= 0.5. For details of plot see Figure S6.

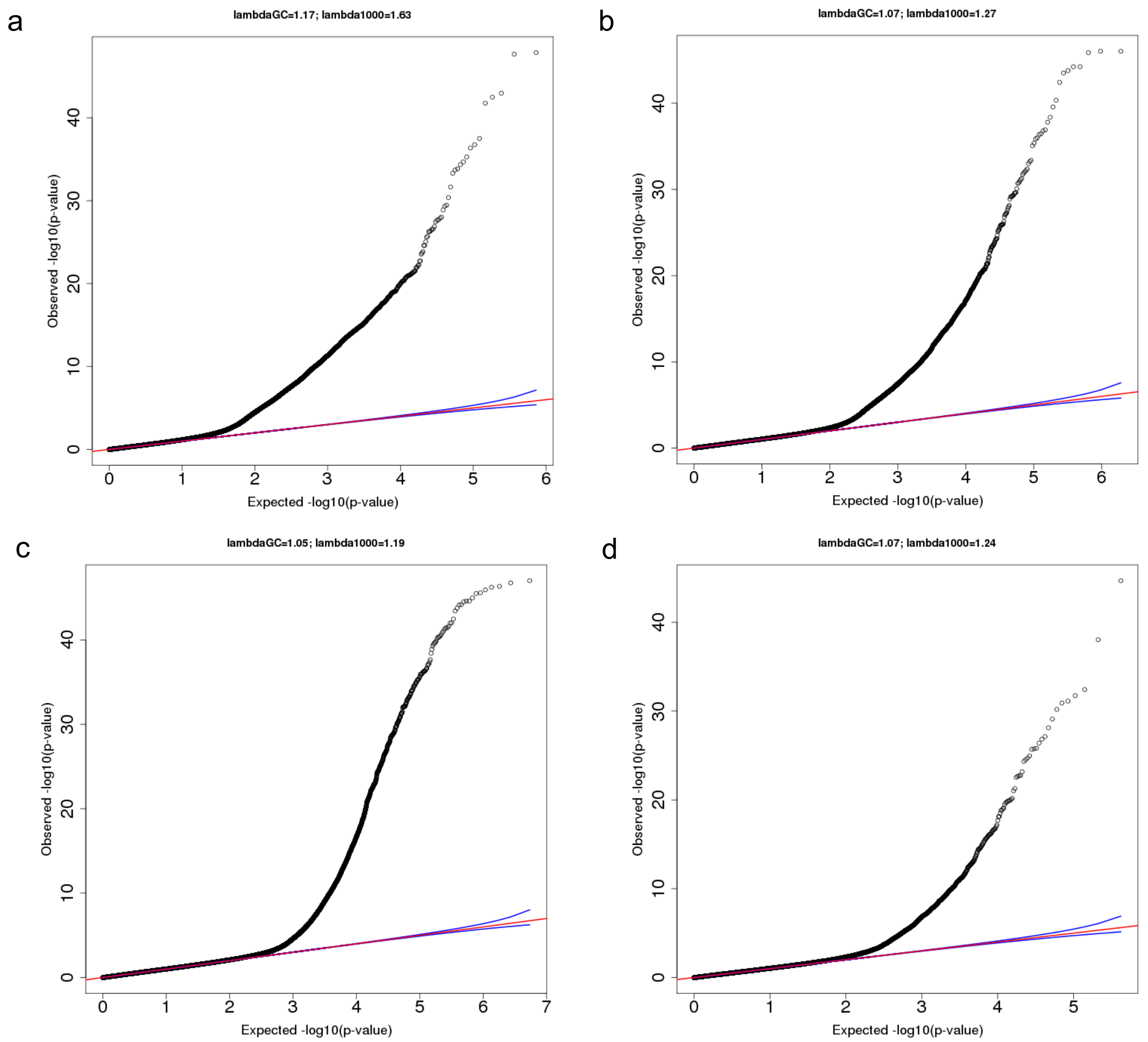

Figure S8. QQ plots for Batch GWAS stratified by GC Content. Percent GC is calculated in a 25 bp window surrounding the sites which are then stratified into a) GC <= 20, b) 20 < GC <= 35, 35 < GC <= 65, GC > 65. For details of plot see Figure S6.

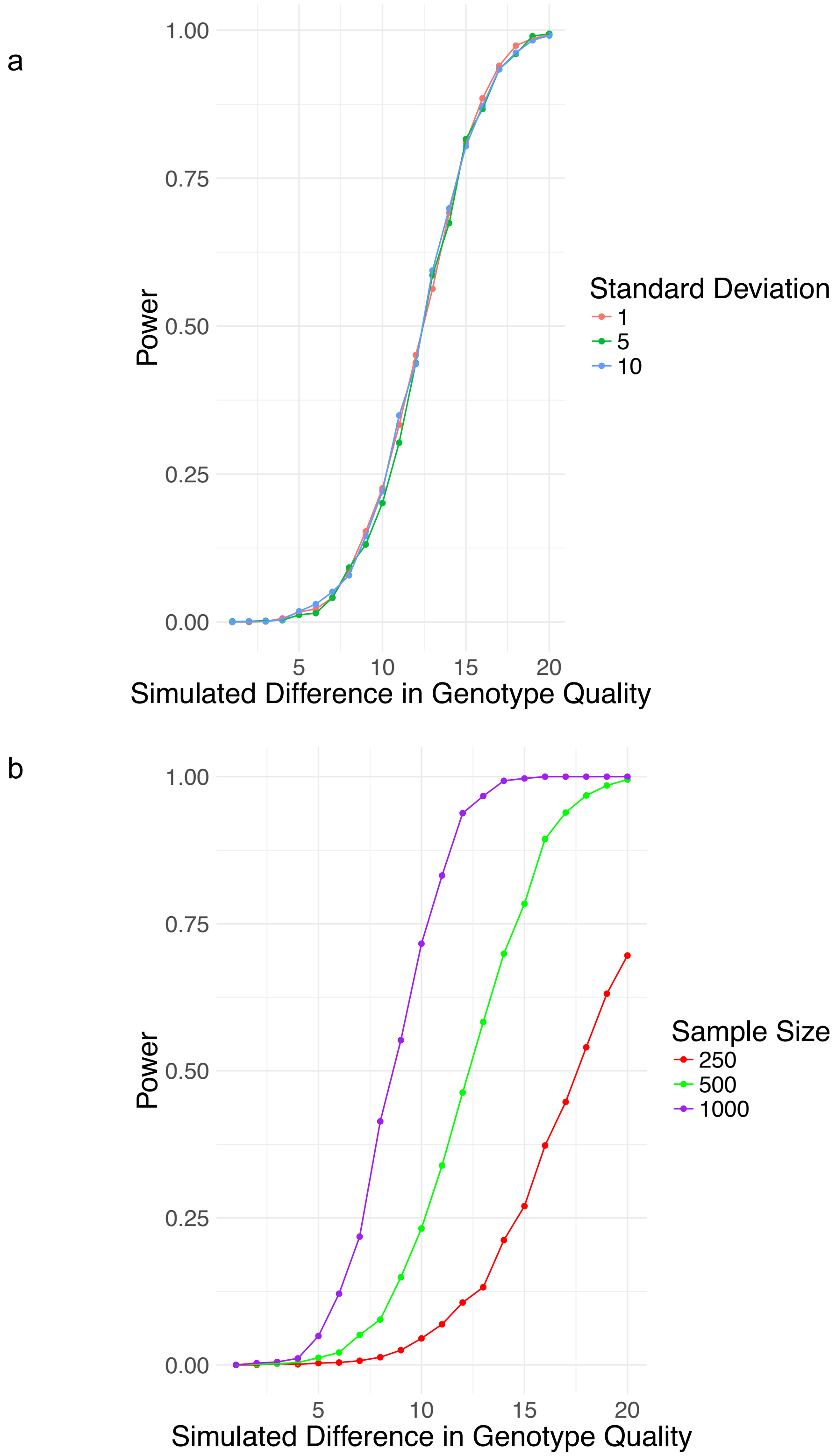

Figure S9. Power calculations for differential genotype quality filter. In a) the standard deviation for the distribution of the genotype quality is varied and in b) the sample size for both groups. For details see Methods.

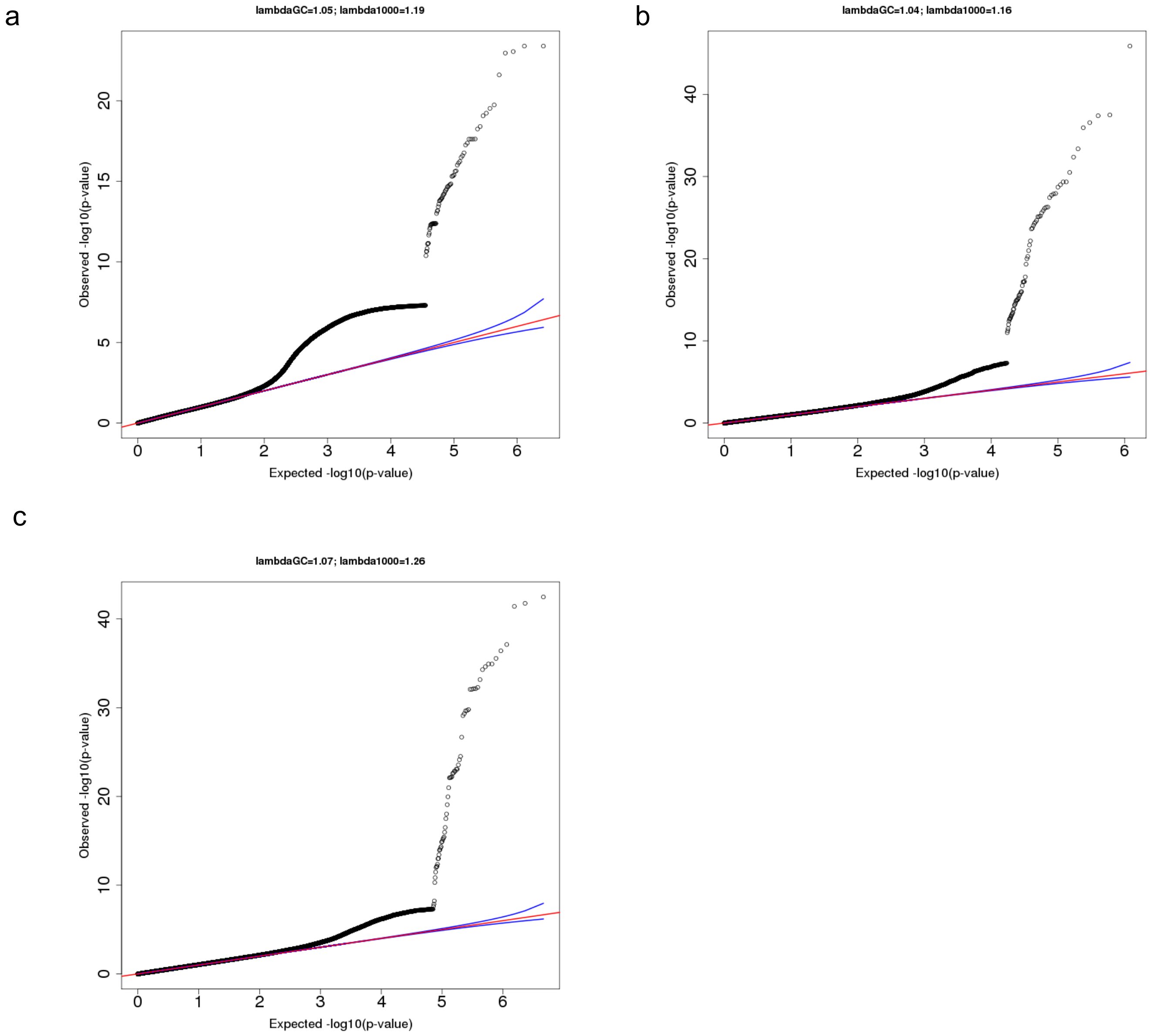

Figure S10. QQ plots for Batch GWAS post filtering stratified by MAF, a) is 0.01 <= MAF <= 0.05, in b) is 0.05 < MAF < 0.1, and c) is 0.1 < MAF <= 0.5.
For details of plot see Figure S6.

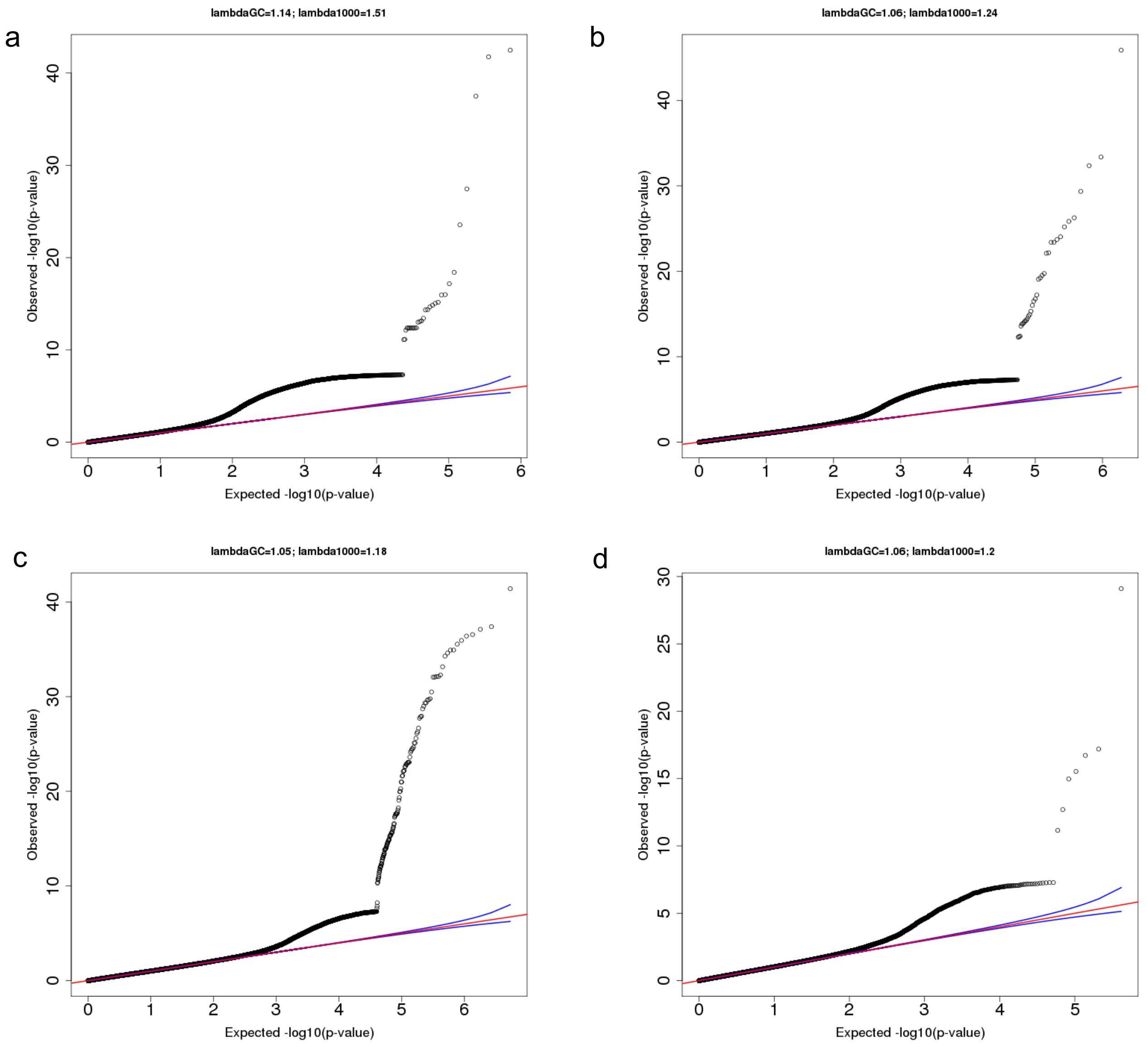

Figure S11. QQ plots for Batch GWAS post filtering stratified by GC Content. Percent GC is calculated in a 25 bp window surrounding the sites which are then stratified into a) GC <= 20, b) 20 < GC <= 35, 35 < GC <= 65, GC > 65. For details of plot see Figure S6.

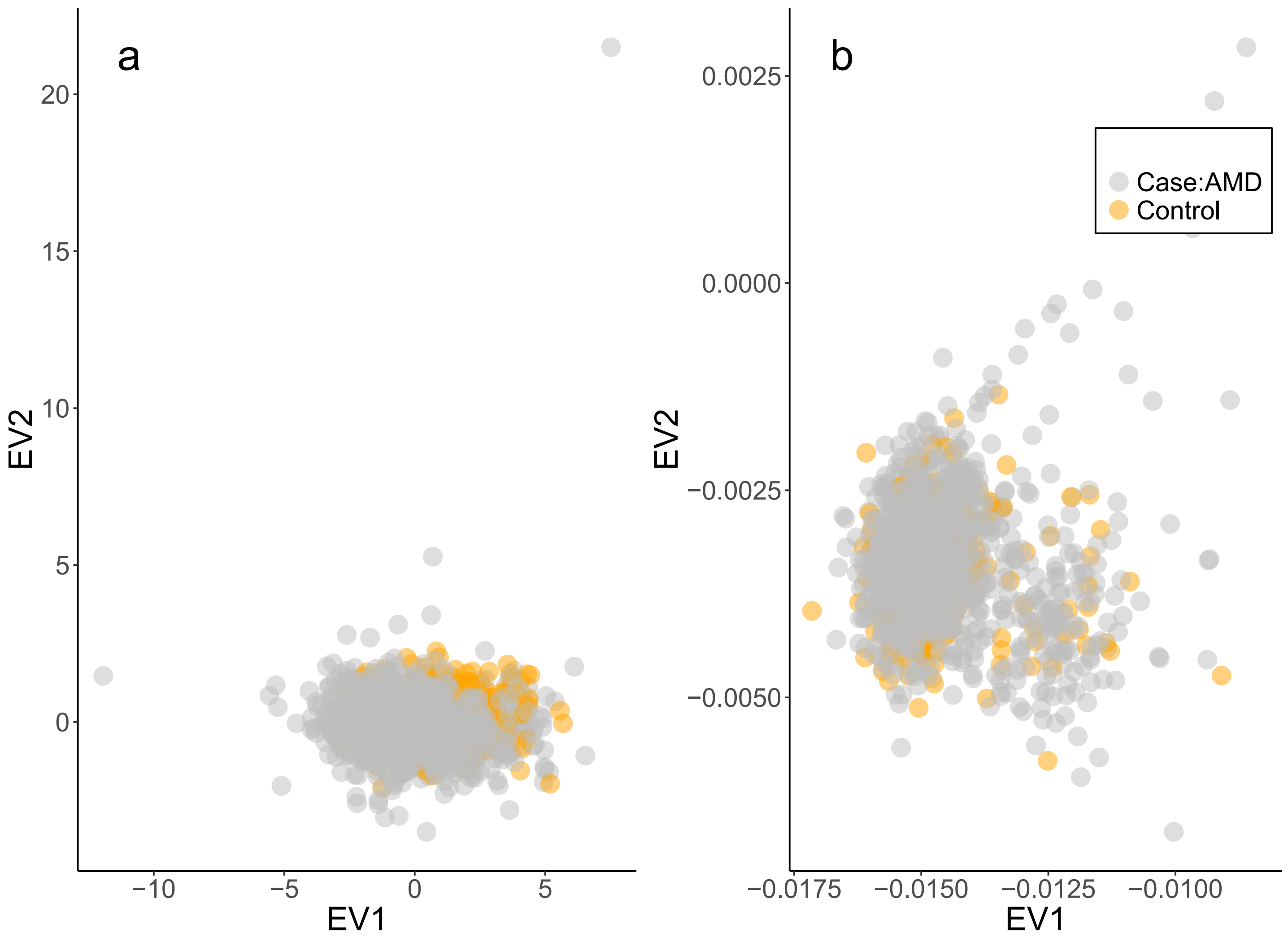

Figure S12. PCA plots for AMD GWAS with no batch effect. In a) are the first two eigenvectors plotted for PCA applied to summary statistics from genotypeeval (see Figure 1 for details). In b) are eigenvectors plotted for PCA applied to 250,000 common SNPs.

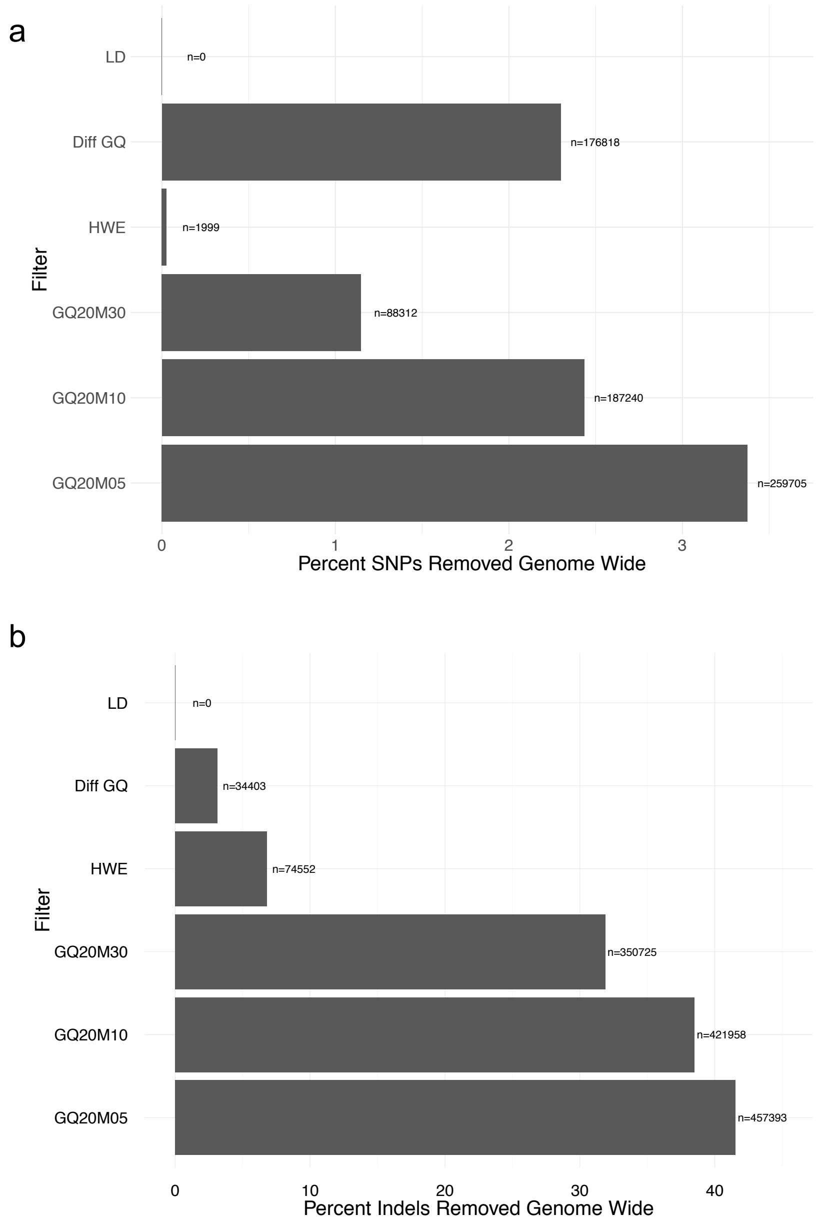

Figure S13. Performance of filters on an Age-Related Macular Degeneration (AMD) GWAS with no batch effect by variant type. Percent (and number, n) of (a) SNPs and (b) Indels removed genome wide in an AMD GWAS with no batch effect where a total of 7,695,436 SNPs and 1,095,989 Indels were analyzed

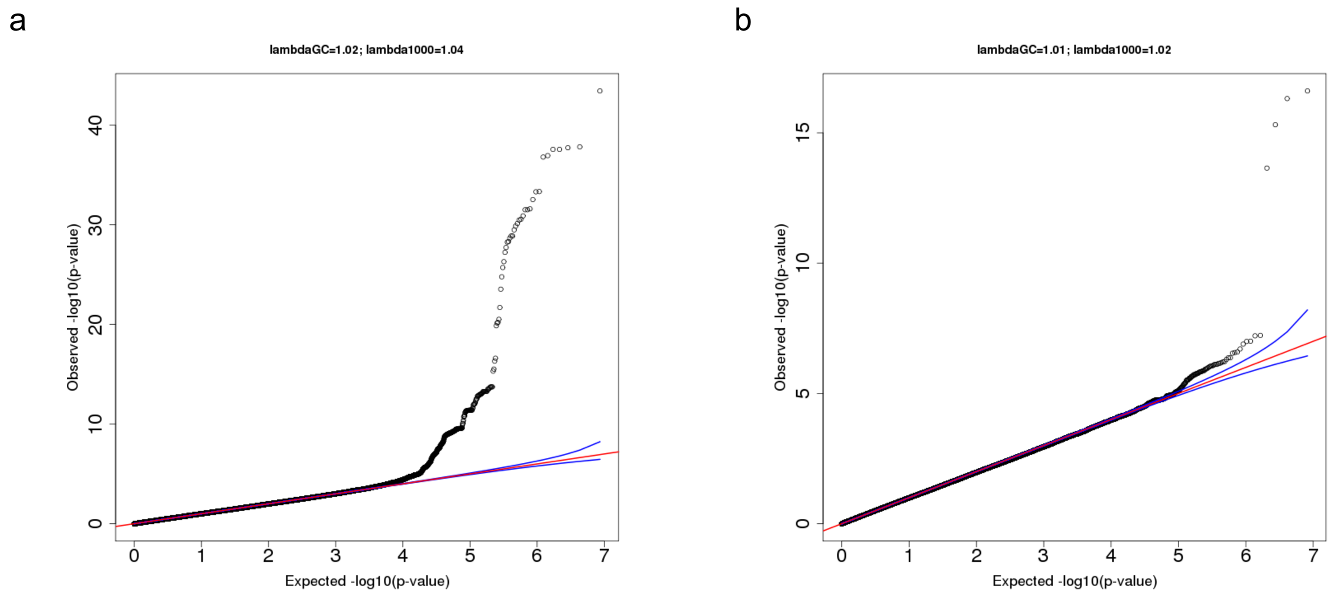

Figure S14. QQ plots for a) AMD No Batch Effect GWAS and b) AMD No Batch Effect GWAS post filtering. lambdaGC is genomic control, lambda 1000 is genomic control with small sample correction, in red is the reference line and in blue are 95% confidence bands for the reference line.

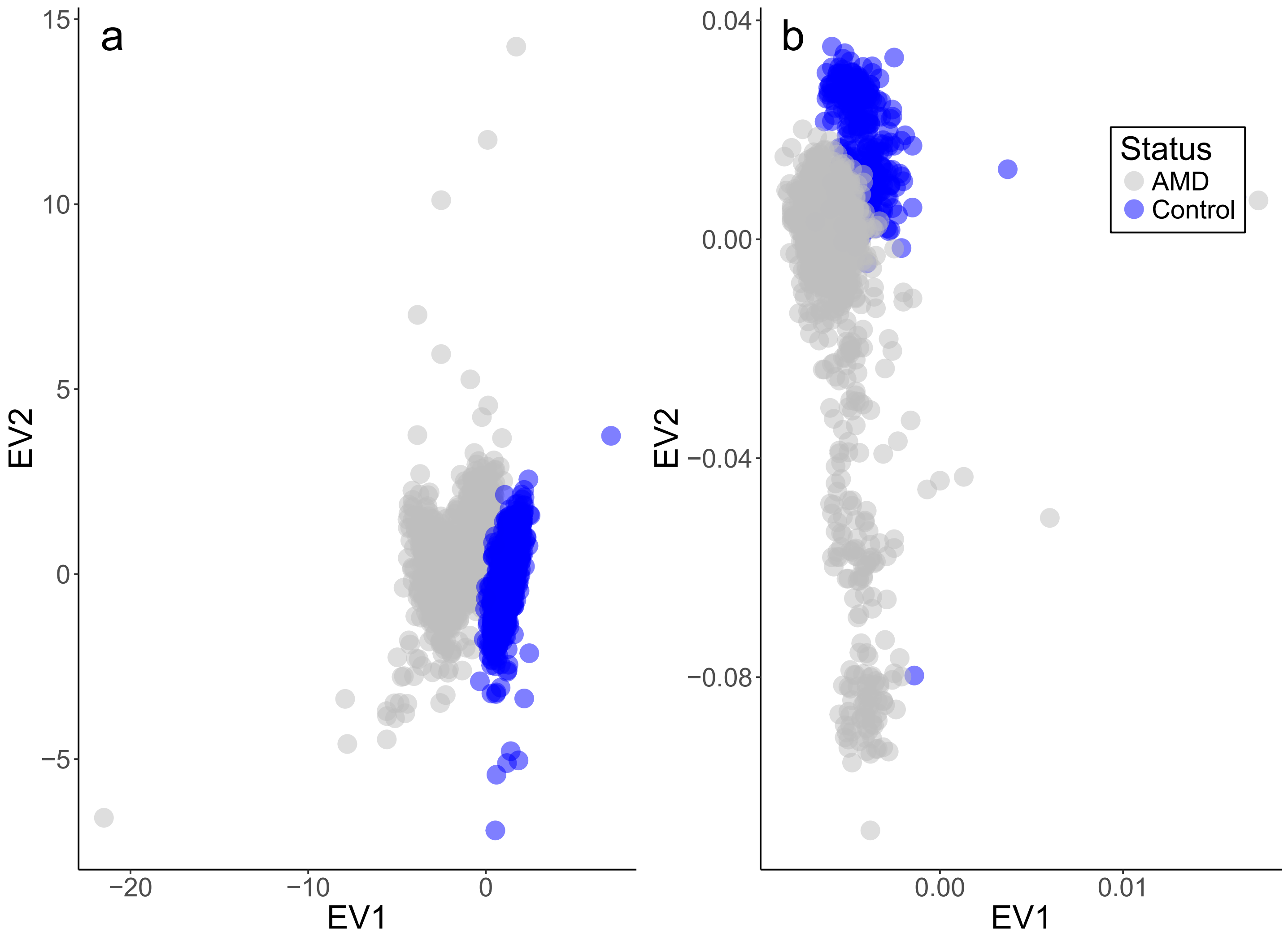

Figure S15. PCA plots for AMD SNP analysis with batch effect. In a) are the first two eigenvectors plotted for PCA applied to summary statistics from genotypeeval (see Figure 1 for details). In b) are eigenvectors plotted for PCA applied to 250,000 common SNPs.

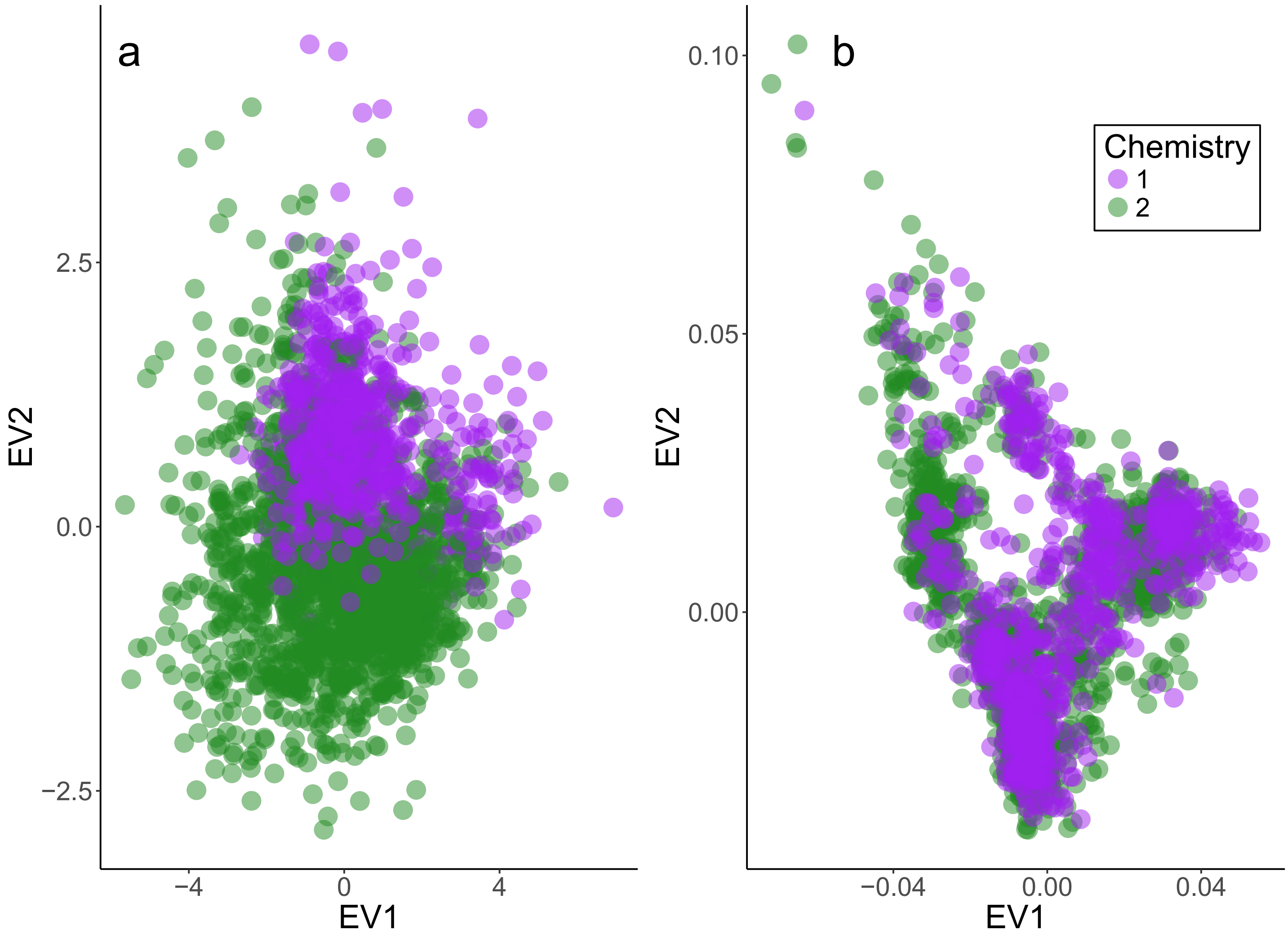

Figure S16. PCA plots for RA GWAS with batch effect. In a) are the first two eigenvectors plotted for PCA applied to summary statistics from genotypeeval (see Figure 1 for details). In b) are eigenvectors plotted for PCA applied to 250,000 common SNPs.

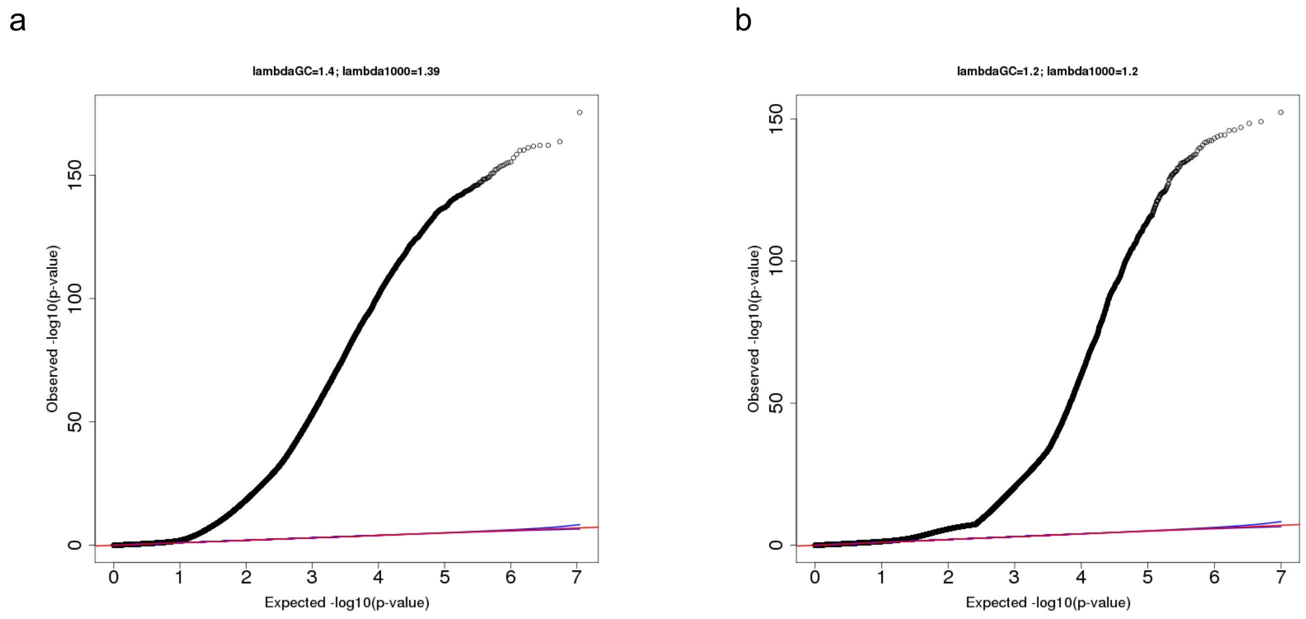

Figure S17. QQ plots for a) RA Batch GWAS and b) RA Batch GWAS post filtering. For details of plot see Figure S6.
