# Supplementary material for: Identifying and mitigating batch effects in whole genome sequencing data
Source: BMC Bioinformatics. 2017 Jul 24;18:351. doi: 10.1186/s12859-017-1756-z (PMC5525370; doi:10.1186/s12859-017-1756-z)
Supplement: Supplementary file 2 — (PDF 112 kb) [file 12859_2017_1756_MOESM2_ESM.pdf]

										Analysis			
**Year**	**N**	**Disease**	**Vendor**	**Platform**	**Library Prep Name**	**Size Exclusion**	**Library Prep PCR**	**Sequencing Flow Cell**	**Clustering Chemistry**	**Batch GWAS**	**AMD No Batch GWAS**	**AMD Batch Candidate Analysis**	**RA Batch GWAS**
**2010**	2	Mixed	BGI	NA	BGI Small Fragment	gel	yes	NA	NA				
**2010**	92	Mixed	Illumina	NA	NA	NA	NA	NA	NA	x		x	
**2011**	332	Mixed	Illumina	HiSeq	Legacy PCR Based	gel	yes	NA	NA	x		x	
**2011**	200	Mixed	deCODE	NA	NA	NA	NA	NA	NA	x		x	
**2011**	160	Mixed	BGI	NA	NA	NA	NA	NA	NA				
**2012**	132	Mixed	Illumina	NA	NA	NA	NA	NA	NA	x		x	
**2013**	138	Mixed	Illumina	HiSeq	Trueseq PCR-Free	SPRI	no	NA	NA	x		x	
**2014**	175	Mixed	Broad	X Ten	Standard PCR+	gel	yes	NA	NA	x		x	
**2015**	279	AD	HLI	X Ten	ILMN TruSeq Nano	SPRI	yes	V2	V1		x		
**2015**	1252	AMD	HLI	X Ten	ILMN TruSeq Nano	SPRI	yes	V2	V1		x	x	
**2015**	770	RA	HLI	X Ten	ILMN TruSeq Nano	SPRI	yes	V2	V1				x
**2016**	1528	RA	HLI	X Ten	Kapa Hyper Prep	SPRI	no	V2.5	V2				x

Table S1. Sequencing vendors and key protocol variables for the samples used in this study. BGI, Beijing Genomics Institute; deCODE, deCODE genetics; Broad, Broad Institute of MIT and Harvard; HLI, Human Longevity Institute, Inc.; SPRI, Solid Phase Reversible Immobilization is gel free size exclusion. The columns under the header Analysis indicate where the samples from that analysis originated. AD is Alzheimers Disease, AMD is Age-Related Macular Degeneration, RA is Rheumatoid Arthritis.

Metric	Filter	Group 1: Mean	Group 1: SD	Group 2: Mean	Group 2: SD	p-value[a]
GATK Genotype Quality	No Masking	91.49	2.65	90.77	3.57	NS
GATK Genotype Quality	Self-Chain	92.07	2.68	91.24	3.63	NS
GATK Genotype Quality	Low Complexity	91.51	2.65	90.82	3.56	NS
GATK Genotype Quality	Centromeres	91.64	2.65	91.49	3.43	NS
GATK Genotype Quality	Blacklist	91.53	2.66	90.85	3.55	NS
GATK Genotype Quality	Genotype Quality < 90	98.83	0.04	98.84	0.07	NS
GATK Genotype Quality	SegDups	92.08	2.7	91.27	3.64	NS
GATK Genotype Quality	Repeat Mask	93.59	2.86	93.22	3.32	NS
Median Read Depth	No Masking	33.65	4.69	35.39	6.81	NS
Median Read Depth	Self-Chain	33.81	4.73	35.4	6.83	NS
Median Read Depth	Low Complexity	33.65	4.69	35.42	6.8	NS
Median Read Depth	Centromeres	33.67	4.69	35.58	6.76	NS
Median Read Depth	Blacklist	33.64	4.69	35.44	6.78	NS
Median Read Depth	Genotype Quality < 90	35.66	4.36	38.02	6.46	0.0004
Median Read Depth	SegDups	33.79	4.73	35.38	6.8	NS
Median Read Depth	Repeat Mask	35.04	4.95	36.78	6.83	NS
Ti/Tv in Non Coding Regions	No Masking	2.01	0.01	1.95	0.02	< 0.0001
Ti/Tv in Non Coding Regions	Self-Chain	2.02	0.01	1.95	0.02	< 0.0001
Ti/Tv in Non Coding Regions	Low Complexity	2.01	0.01	1.95	0.02	< 0.0001
Ti/Tv in Non Coding Regions	Centromeres	2.02	0.01	1.98	0.01	< 0.0001
Ti/Tv in Non Coding Regions	Blacklist	2.01	0.01	1.95	0.02	< 0.0001
Ti/Tv in Non Coding Regions	Genotype Quality < 90	2.04	0.02	2.03	0.01	< 0.0001
Ti/Tv in Non Coding Regions	SegDups	2.03	0.01	1.97	0.02	< 0.0001
Ti/Tv in Non Coding Regions	Repeat Mask	2	0.01	1.98	0.01	< 0.0001
Ti/Tv in Coding Regions	No Masking	2.99	0.04	2.9	0.03	< 0.0001
Ti/Tv in Coding Regions	Self-Chain	3.13	0.05	3.05	0.04	< 0.0001
Ti/Tv in Coding Regions	Low Complexity	2.99	0.04	2.91	0.03	< 0.0001
Ti/Tv in Coding Regions	Centromeres	2.99	0.04	2.9	0.03	< 0.0001
Ti/Tv in Coding Regions	Blacklist	2.99	0.04	2.9	0.03	< 0.0001
Ti/Tv in Coding Regions	Genotype Quality < 90	3.11	0.06	3.01	0.04	< 0.0001
Ti/Tv in Coding Regions	SegDups	3.09	0.05	3.01	0.04	< 0.0001
Ti/Tv in Coding Regions	Repeat Mask	3	0.04	2.92	0.03	< 0.0001
% Confirmed in 1000 Genomes	No Masking	0.81	0.01	0.77	0.01	< 0.0001
% Confirmed in 1000 Genomes	Self-Chain	0.84	0.01	0.8	0.01	< 0.0001
% Confirmed in 1000 Genomes	Low Complexity	0.81	0.01	0.77	0.01	< 0.0001
% Confirmed in 1000 Genomes	Centromeres	0.81	0.01	0.78	0.01	< 0.0001
% Confirmed in 1000 Genomes	Blacklist	0.81	0.01	0.77	0.01	< 0.0001
% Confirmed in 1000 Genomes	Genotype Quality < 90	0.85	0.01	0.81	0.01	< 0.0001
% Confirmed in 1000 Genomes	SegDups	0.84	0.01	0.81	0.01	< 0.0001
% Confirmed in 1000 Genomes	Repeat Mask	0.89	0	0.87	0	< 0.0001
Percent Heterozygote	No Masking	0.08%	0.00%	0.08%	0.00%	< 0.0001
Percent Heterozygote	Self-Chain	0.08%	0.00%	0.08%	0.00%	< 0.0001
Percent Heterozygote	Low Complexity	0.08%	0.00%	0.08%	0.00%	< 0.0001
Percent Heterozygote	Centromeres	0.08%	0.00%	0.08%	0.00%	< 0.0001
Percent Heterozygote	Blacklist	0.08%	0.00%	0.08%	0.00%	< 0.0001
Percent Heterozygote	Genotype Quality < 90	0.07%	0.00%	0.08%	0.00%	< 0.0001
Percent Heterozygote	SegDups	0.08%	0.00%	0.08%	0.00%	< 0.0001
Percent Heterozygote	Repeat Mask	0.09%	0.00%	0.09%	0.01%	NS

Table S2. Mean and standard deviation by batch of metrics post filtering.

[a] P-value using a Wilcoxon Rank Sum Test, two-sided alternative, with a Bonferroni adjustment for multiple tests.

	Not GWS	GWS	Total	% UGA	lambda_GC[a]	lambda_1000
**Overall**	8453473	7370	8460843	0.09%	1.07	1.25

Stratified

GC[b] Content	Not GWS	GWS	Total	% UGA	lambda_GC	lambda_1000
GC <= 0.2	729103	2562	731665	0.35%	1.17	1.63
0.2 < GC <= 0.35	1910776	2012	1912788	0.11%	1.07	1.27
0.35 < GC <= 0.65	5393001	2444	5395445	0.05%	1.05	1.19
GC > 0.65	420593	352	420945	0.08%	1.07	1.24

Stratified

MAF	Not GWS	GWS	Total	% UGA	lambda_GC	lambda_1000
MAF >= .01 & <= 0.05	2622461	4755	2627216	0.18%	1.07	1.24
MAF > 0.05 & <= 0.1	1204987	1117	1206104	0.09%	1.05	1.18
MAF > 0.1 & <= .5	4626025	1498	4627523	0.03%	1.07	1.27

Table S3. Summary of Batch GWAS, overall and stratified by GC content and Minor Allele Frequency (MAF). Reporting the number of sites not genome wide significant (GWS), GWS, and total number of sites tested. lambda_GC is the genomic control and lambda_1000 is genomic control correcting for small sample size. UGA is unconfirmed genome-wide significant association.

[a] GC refers to genomic control

[b] GC refers to % GC content in 25 BP window surrounding association

	UGAs (n=7370)		UGAs: SNP (n=1901)		UGAs: Indel (n=5469)	
**Filter**	**Filtered**	**Percent Filtered**	**Filtered**	**Percent Filtered**	**Filtered**	**Percent Filtered**
**LD**	5365	72.8	1335	70.22	4030	73.69
**GQ20M05**	7188	97.53	1816	95.53	5372	98.23
**GQ20M10**	5495	74.56	1522	80.06	3973	72.65
**GQ20M30**	4897	66.45	1298	68.3	3599	65.81
**Diff GQ**	5487	74.45	1273	66.96	4214	77.05
**Centromeres**	63	0.85	26	1.37	37	0.68
**Self-Chain**	459	6.23	196	10.31	263	4.81
**Blacklist**	51	0.69	24	1.26	27	0.49
**Low Complexity**	31	0.42	12	0.63	19	0.35
**Segmental Duplications**	720	9.77	253	13.31	467	8.54
**Repeat Mask**	3708	50.3	981	51.6	2727	49.86

Table S4. Filtering false positive associations from the Batch-GWAS. GQ20Mx filter, filter sites with more than x% missingness after setting genotypes with GQ < 20 to missing; Diff GQ, differential genotype quality filter; Diff Miss, differential missingness filter; LD, linkage disequilibrium. UGA is unconfirmed genome-wide significant association.

	UGAs (n=205)		UGAs: SNP (n=74)		UGAs: Indel (n=131)	
**Filter**	**Filtered**	**Percent Filtered**	**Filtered**	**Percent Filtered**	**Filtered**	**Percent Filtered**
**GQ20M05**	170	82.93	58	78.38	112	85.5
**GQ20M10**	70	34.14	36	48.65	34	25.95
**Centromeres**	3	1.46	0	0	3	2.29
**Self-Chain**	28	13.66	11	14.86	17	12.98
**Blacklist**	2	0.98	0	0	2	1.53
**Low Complexity**	5	2.44	4	5.41	1	0.76
**Segmental Duplications**	30	14.63	12	16.22	18	13.74
**Repeat Mask**	96	46.83	37	50	59	45.04

Table S5. Filtering false positive associations from the Batch-GWAS after applying recommended filters. For details see Table S4.

**Filtered**	Not GWS	GWS	Total	% UGA	lambda_GC[a]	lambda_1000	% UGA Filtered
Overall	8407062	205	8460843	0.00002	1.06	1.22	97.22%

Stratified

GC[b] Content	Not GWS	GWS	Total	% UGA	lambda_GC	lambda_1000	% UGA Filtered
GC <= 0.2	712126	30	712156	0.00004	1.14	1.51	98.83%
0.2 < GC <= 0.35	1901230	34	1901264	0.00002	1.06	1.24	98.31%
0.35 < GC <= 0.65	5379434	134	5379568	0.00002	1.05	1.18	94.52%
GC > 0.65	414272	7	414279	0.00002	1.06	1.2	98.01%

Stratified

MAF	Not GWS	GWS	Total	% UGA	lambda_GC	lambda_1000	% UGA Filtered
MAF >= .01 & <= 0.05	2596184	72	2596256	0.00003	1.05	1.19	98.49%
MAF > 0.05 & <= 0.1	1197220	69	1197289	0.00006	1.04	1.16	93.82%
MAF > 0.1 & <= .5	4613658	64	4613722	0.00001	1.07	1.26	95.73%

Table S6. Summary of Batch GWAS post filtering, overall and stratified by GC content and Minor Allele Frequency (MAF). Reporting the number of sites not genome wide significant (GWS), GWS, and total number of sites tested. lambda_GC is the genomic control and lambda_1000 is genomic control correcting for small sample size. UGA is unconfirmed genome-wide significant association.

[a] GC refers to genomic control

[b] GC refers to % GC content in 25 BP window surrounding association

CHR	Position[a]	Gene	Power	Power
			1218 cases, 250 controls	1252 cases, 678 controls
**1**	**196710325**	**CFH**	**0.53878**	**0.9974**
3	64719689	ADAMTS9	0	0.00004
3	99762695	COL8A1-FILIP1L	0.00004	0.00044
4	109669323	CFI	0.00002	0.00023
6	30806580	IER3-DDR1	0.00001	0.0001
6	31962685	C2-CFB	0.00207	0.05585
6	43858890	VEGFA	0.00003	0.00047
6	116122572	COL10A1	0	0.00003
8	23225458	TNFRSF10A	0.00004	0.0005
9	99146083	TGFBR1	0.00001	0.00006
**10**	**122454932**	**ARMS2-HTRA1**	**0.9997**	**1**
13	31245188	B3GALTL	0	0.00004
14	68318360	RAD51B	0.00001	0.00005
15	58396268	LIPC	0.00002	0.00019
16	56963321	CETP	0.00006	0.00097
19	6718376	C3	0.0133	0.20278
19	44919689	APOE	0.00009	0.00174
22	32663679	TIMP3	0.00043	0.00991
22	38080269	SLC16A8	0.00002	0.00017

Table S7. Power calculations for AMD GWAS and Candidate SNP Analysis. [a] Sites are reported in GRCh38 coordinates.

	All			Type I Error			Confirmed Associations			Unconfirmed Associations		
	**SNPs**	**Indels**	**Total**	**SNPs**	**Indels**	**Total**	**SNPs**	**Indels**	**Total**	**SNPs**	**Indels**	**Total**
**N**	7695436	1095989	8636121				220	0	220	16	31	47
**Diff GQ**	176818	34403	211221	0.024	0.031	0.020	15	0	15	15	8	23
**GQ20M05**	259705	315726	575431	0.067	0.288	0.030	1	0	1	1	3	4
**GQ20M10**	187240	284213	471453	0.055	0.259	0.022	1	0	1	1	0	1
**GQ20M30**	88312	216098	304410	0.035	0.197	0.010	0	0	0	1	0	1
**LD**	0	0	0	0.000	0.000	0.000	0	0	0	0	0	0
**HWE**	1999	791	2790	0.000	0.001	0.000	0	0	0	0	0	0
**LD + GQ20M30 + Diff GQ**	261833	246571	508404	0.034	0.225	0.059	15	0	15	15	8	23

Table S8. Number of variants broken down by type eliminated by each filter in the AMD GWAS with no batch effect. Confirmed Associations, P < 5E-8 in known AMD loci; Unconfirmed Associations, P < 5E-8 sites not in known AMD loci.

	Not GWS	GWS	Total	% UGA	lambda_GC[a]	lambda_1000
Overall	10643204	381139	11024343	3.46%	1.4	1.39
After Filtering	9983190	38733	10021923	0.39%	1.2	1.2

Table S9. Summary of RA GWAS with batch effect, pre and post filtering. Reporting the number of sites not genome wide significant (GWS), GWS, and total number of sites tested. lambda_GC is the genomic control and lambda_1000 is genomic control correcting for small sample size. UGA is unconfirmed genome-wide significant association.

[a] GC refers to genomic control

Indels	UGA				
Difficult to sequence region	N not in Region	N in Region	N Total	% In Region	% of Genome Region Covers
Segmental Duplications	304560	29738	334298	8.9	13.65
Low Complexity	331606	2692	334298	0.8	0.21
Centromeres	333997	301	334298	0.1	2.01
Blacklist	333823	475	334298	0.1	0.39
Repeat Mask	55930	278368	334298	83.3	53.02
Self-Chain	320000	14298	334298	4.3	6.02

SNPs	UGA				
Difficult to sequence region	N not in Region	N in Region	N Total	% In Region	% of Genome Region Covers
Segmental Duplications	42786	4055	46841	8.7	13.65
Low Complexity	46129	712	46841	1.5	0.21
Centromeres	46553	288	46841	0.6	2.01
Blacklist	46753	88	46841	0.2	0.39
Repeat Mask	6135	40706	46841	86.9	53.02
Self-Chain	45423	1418	46841	3	6.02

Table S10. Number and Percent of unconfirmed associations from RA (Rheumatoid Arthritis) GWAS with Batch Effect that lie in Difficult to Sequence Regions of Genome.

	UGA (n=381,139)		
**Filter**	**Filtered**	**Percent Filtered**	**N (post filtering)**
**LD**	18452	4.84%	11005891
**GQ20M30**	65705	17.24%	10477130
**Diff GQ**	332717	87.30%	10423858
**LD + GQ20M30 + Diff GQ**	342406	89.84%	9983190
**No Filtering**			11024343
	**UGA: SNP (n=46,841)**		
**Filter**	**Filtered**	**Percent Filtered**	**N**
**LD**	6468	13.81%	8073877
**GQ20M30**	11600	24.76%	7971170
**Diff GQ**	40420	86.29%	8016842
**LD + GQ20M30 + Diff GQ**	42254	90.21%	7920020
**No Filtering**			8080345
	**UGA: Indel (n=334,298)**		
**Filter**	**Filtered**	**Percent Filtered**	**N**
**LD**	11984	3.58%	2932014
**GQ20M30**	54105	16.18%	2505960
**Diff GQ**	292297	87.44%	2407016
**LD + GQ20M30 + Diff GQ**	300152	89.79%	2063170
**No Filtering**			2943998

Table S11. Results from filtering out batch effects from Rheumatoid Arthritis GWAS with Batch Effect.
